# Extracellular vesicle bioactivity and potential for clinical development are determined by mesenchymal stromal cell clonal subtype

**DOI:** 10.1186/s13287-025-04665-z

**Published:** 2025-10-16

**Authors:** Savvas Ioannou, Alasdair G. Kay, Andrew P. Stone, Emma Rand, Samuel Elberfeld, William Bolton, Tony Larson, Rachel E. Crossland, Oksana Kehoe, David A. Mentlak, Xiao-Nong Wang, Chris MacDonald, Paul G. Genever

**Affiliations:** 1 York Biomedical Research Institute, Department of Biology, University of York, York, UK; 2 Translational and Clinical Research Institute, Newcastle University, Newcastle upon Tyne, UK; 3 Centre for Regenerative Medicine Research, School of Medicine, Keele University, Keele, Staffordshire UK; 4 Robert Jones and Agnes Hunt Orthopaedic Hospital, Oswestry, Shropshire UK; 5 Mesenbio Ltd, Department of Biology, University of York, York, UK

**Keywords:** Mesenchymal stromal cells, Heterogeneity, Extracellular vesicles, Matrix proteins, Biotherapeutic, Arthritis

## Abstract

**Background:**

Mesenchymal stromal cells (MSCs) have been used in numerous clinical trials but very few reach phase 3 or market authorisation. Progress is often hampered by the use of non-clonal, heterogeneous and uncharacterised MSC cultures and lack of mechanistic understanding. There is limited evidence of MSC engraftment in vivo and disease resolution may be the result of the paracrine effects of the MSC secretome, rather than the cells per se. Extracellular vesicles (EVs) are key components of the MSC secretome and there is growing interest in the use of EVs as cell-free therapies. However, like MSCs, heterogeneity can exist within any therapeutic EV pool. Here we used immortalised clonal MSC lines, termed Y201 and Y202, to examine how MSC phenotype influences EV character and function.

**Methods:**

EVs were isolated by ultracentrifugation and characterised by nano-sizing, ultrastructural morphometric analysis, western blotting, mass spectrometry and miRNA screening. Bioactivity was determined by phosphorylation of ERK1/2, proliferation and T cell polarisation assays and using two in vivo models of inflammatory disease.

**Results:**

EVs from Y201 and Y202 MSCs were morphologically similar, however, Y201 EVs were more abundant in EV biomarkers versus Y202 EVs, with an enhanced miRNA and proteomic content. Computational analysis of the Y201 EV proteome identified significant enrichment in matrix-associated proteins, predicted to contribute to an elaborate EV corona particularly abundant in RGD-containing proteins fibronectin and MFG-E8, which was confirmed by western blotting. Y201 EVs, but not Y202 EVs, significantly increased the proliferation of articular chondrocytes in a dose-dependent manner, and the proliferative effect of Y201 EVs was mediated at least in part via an RGD (integrin)-FAK-ERK1/2 axis. Both Y201 and Y202 EV subsets significantly reduced proliferative index scores of activated T cells. However, only Y201 EVs, not Y202 EVs, suppressed disease activity compared to controls in different in vivo models of inflammatory peritonitis and arthritis.

**Conclusions:**

EVs released by closely related MSC subtypes within the same heterogeneous population differ significantly in terms of cargo abundance, bioactivity, and pre-clinical in vivo efficacy. Analysis of defined EV subsets will aid mechanistic understanding and prioritisation for EV therapeutics.

**Supplementary Information:**

The online version contains supplementary material available at 10.1186/s13287-025-04665-z.

## Background

Mesenchymal stromal cells (MSCs, often referred to as mesenchymal stem cells) can exhibit trilineage differentiation capacity (osteogenic, chondrogenic and adipogenic) and immuno-suppressive properties [1–3]. Largely based on these biological functions, MSCs have been used in numerous clinical trials targeting musculoskeletal and inflammatory disorders but the range of clinical indications is very broad and success is variable [4–8]. Although phase 1 and 2 clinical trials continue to rise, very few reach phase 3 [4, 7]. Progress has been hampered largely by the use of non-clonal, heterogeneous and uncharacterised MSC cultures and lack of mechanistic understanding [5]. In addition, there is limited evidence of MSC engraftment in vivo [9, 10], and disease resolution may be the direct result of MSC apoptosis [11] and/or the paracrine effects of the MSC secretome, rather than the cells per se [12–17].

Key components of the MSC secretome are extracellular vesicles (EVs). The term EV describes a heterogeneous population of secreted membrane vesicles [18] that have the ability to exchange biological components between cells, thereby acting as signalling vehicles [19–21]. There are different types of EVs, frequently categorised as exosomes, microvesicles (MVs), and apoptotic bodies based on their secretory process and size [18, 22]. Exosomes have a typical diameter of 30–150 nm [23] and are formed during the maturation of multivesicular bodies (MVBs). Biogenesis of intraluminal vesicles occurs by the inward budding of the limiting membrane of the MVB followed by scission [24, 25]. Intraluminal vesicles are released as exosomes into extracellular space during the fusion of the MVB with the plasma membrane [26]. In contrast, MVs are formed by the outward budding of the plasma membrane [27] ranging in size from 100 nm to 1000 nm in diameter [23]. Apoptotic bodies are larger membrane-bound fragments (50–5000 nm in diameter) that are generated as cells undergo apoptosis [23]. Apoptotic bodies are no longer considered the inert debris of dying cells, indeed they have important functions in many aspects of tissue homeostasis [28]. During EV formation, EVs are packaged with proteins, mRNAs and miRNAs and delivered to the recipient cells by various docking and uptake mechanisms to exert a biological effect [26].

There is growing interest in the use of EVs as cell-free therapies. EVs may be able to offer a more cost-effective, accessible route to clinic compared to their parent cells, with improved safety profiles and amenable transport, storage and administration options. EVs have demonstrated efficacy in several preclinical animal models, including myocardial ischaemia [29], kidney damage [30], acute lung injury [31] and inflammatory arthritis [32] amongst others. However, in a manner similar to MSC therapy, heterogeneity within any therapeutic EV pool will hamper clinical development.

Here we used immortalised clonal MSC lines, termed Y201 and Y202, to examine how the source MSC phenotype influences EV character and function. In previous work, we isolated Y201 and Y202 from the same donor and performed of in-depth phenotypic characterisation. We demonstrated that both Y201 and Y202 identify as “MSCs” by surface protein expression and independent transcriptomic profiling [33, 34]. Y201 MSCs lack expression of CD317 (CD317^neg^) and are model mesenchymal *stem* cells with strong trilineage differentiation and immune-suppressive properties. Y202 (CD317^pos^) MSCs have weak differentiation capacity and appear to function primarily as immune-regulatory cells [33–35]. Our findings demonstrate that EVs released by closely related MSC subtypes within the same heterogeneous cell population differ significantly in terms of cargo abundance, bioactivity and potential for clinical efficacy, which will advance our understanding of EV biology and parent cell selection for EV therapeutics.

## Methods

### Cell culture

Y201 and Y202 MSCs were cultured using Dulbecco’s Modified Eagle’s Medium (DMEM) (ThermoFisher Scientific, Cat: 41966) supplemented with 10% foetal bovine serum (FBS) and 1% Penicillin/Streptomycin (P/S) (ThermoFisher Scientific Cat: 15140). Human articular chondrocytes (AC) were isolated from primary donors following fully informed ethical consent (LREC 07/Q1105/9). ACs were cultured in DMEM-F12 (ThermoFisher Scientific Cat: 11320-033), supplemented with 10% FBS and 1% P/S.

### Preparation of EV depleted medium

DMEM supplemented with 20% FBS and 1% P/S was centrifuged at 100,000*g* for 18 h at 4 °C to deplete the medium of serum-derived bovine EVs. During cell expansion, medium was diluted in equal volumes with DMEM supplemented with 1% P/S. Cells were grown at 37 °C in 5% CO_2_/95% atmospheric air.

### EV isolation by sequential differential ultracentrifugation

For EV isolation, 5 × 10^5^ cells were seeded in T175 flasks with EV depleted medium and allowed to reach 80–90% confluence. Medium was aspirated and cells were washed three times with PBS and serum-free medium was added. Two collections of conditioned medium were performed every 24 h and stored at – 70 °C, at the end of the second collection, cells were counted. EVs were isolated according to the Thery et al. protocol with minor modifications, all steps were performed at 4 °C [36]. Conditioned media were centrifuged at 300*g* for 5 mins to remove debris and then 2000*g* for 20 min. Supernatant was transferred to Ty45i thick-walled ultracentrifuge tubes (Beckman-Coulter Cat: 355655) and centrifuged at 10,000*g* for 45 min in L100-XP Beckman-Coulter ultracentrifuge to pellet the “10K” EV fraction. The supernatant was transferred to a new Ty45i thick-walled tube and centrifuged at 100,000*g* for 90 min to generate the “100K” EV fraction. Both 10K and 100K fractions were re-suspended in PBS by vigorous pipetting and transferred into thick-walled micro-ultracentrifuge tubes (Beckman-Coulter Cat: 357448) and centrifuged again at 10,000*g* and 100,000*g* in a Beckman-Coulter TL100 ultracentrifuge for 45 and 90 min respectively. The final 10K and 100K EV fractions were resuspended in PBS and transferred to protein LoBind tubes.

### Analysis of EVs using nanoparticle tracking analysis (NTA)

EVs were analysed using the Nanosight LM14 equipped with a green laser (532 nm). The 10K and 100K EV fractions were diluted in PBS to a concentration of 20–150 particles per frame. A script was used obtaining 5 × 60 s video recordings of all events for further analysis. The experimental conditions were as follows: (i) Measuring time: 5 × 60 s, (ii) Blur: Auto, (iii) Detection Threshold: 4–5, (iv) Blur size: Auto, iv) Number of frames: 1499.

### Transmission electron microscopy (TEM)

EV suspension (10 µl) was deposited on a 200 mesh copper grid with a formvar/carbon support film, left for 3 min to air dry, then washed with dH_2_O. Samples were exposed to 1% uranyl-acetate and imaged using a ThermoFisher Tecnai 12 at 120 kV at 18.5 K and 49 K magnification.

### Treatment of Y202 MSCs with focal adhesion kinase inhibitor (FAKi)

Y202 MSCs were seeded on a 6-well plate at a 20,000 cells/cm^2^ using DMEM supplemented with 10% FBS and 1% P/S and left to adhere for 6 h. Cells were washed three times with PBS and serum-free DMEM was added overnight. The following day, cells were treated with 10µΜ focal adhesion kinase inhibitor (PF573228) for 1 h and Y201 EVs were added for 30 min, then washed with PBS to remove any unbound EVs. To generate cell lysates, cells were washed with ice-cold PBS and radioimmunoprecipitation assay (RIPA) buffer supplemented with protease inhibitors (Merck; Cat: 11836153001). Cells were scraped and passed through a 25G needle before centrifuging at 10,000*g* for 20 min. The supernatant was collected and a bicinchoninic acid assay (BCA; ThermoFisher Scientific, Cat: 23225) was performed to quantify the total amount of protein.

### Western blotting

Cell lysates and EVs were separated on a 12% SDS-PAGE gel and transferred to a nitrocellulose membrane using the iBlot2 dry blotting system. Membranes were blocked for an hour with 5% Bovine Serum Albumin (BSA) in PBS-0.1% Tween buffer or Tris Buffered Saline (TBS)-0.1% Tween buffer, the membranes were incubated with the following mouse monoclonal antibodies: (i) Flotillin-1 (1:500; SantaCruz: 133153), (ii) CD63 (1:1000; SantaCruz: 365604), (iii) CD81 (1:1000; SantaCruz: 23962), (iv) Alix (SantaCruz: 166952), (v) GRP78 BiP (Abcam: 21685), v) Fibronectin (1:500; Abcam:2413), (vi) MFG-E8 (1:200; Proteintech: 67797-1-Ig) (vii) ERK1/2 (1:1000; CellSignaling Technology: 4695), (viii) pERK1/2 (1:1000, CellSignaling Technology: 9101) for an hour at room temperature or overnight at 4 °C. After the incubation, membranes were incubated with anti-mouse or anti-rabbit horseradish peroxidase-conjugated secondary antibodies in 1:1500 or 1:2000 dilution respectively. The protein bands were visualised following addition of ECL PicoPlus Chemiluminescent substrate (Cat: 34577, ThermoFisher Scientific) and captured using the iBright imaging system (ThermoFisher Scientific).

### Proteomic analysis of Y201 and Y202 EVs

EVs isolated from 8xT175 of cells were added to 8 M urea with 20 mM HEPES and a phosphatase inhibitor cocktail comprising 1mM sodium orthovanadate, 1 mM β-glycerophosphate and 2.5 mM sodium pyrophosphate. Samples were reduced with 5 mM dithiothreitol at 55 °C for 30 min before alkylating with 15 mM iodoacetamide for 30 min at room temperature. Solutions were diluted to 2 M urea with aqueous 50 mM ammonium bicarbonate before digesting with the addition of 0.5 mg of sequencing grade trypsin/Lys-C protease mixture (Promega. Cat: V5071) and incubated at 37 °C. Digestion was stopped after 16 h with the addition of trifluoroacetic acid to 0.1% (v/v). Resulting peptides were analysed over 1-h LC-MS acquisitions using an Orbitrap Fusion (Thermofisher). Peptides were eluted into the mass spectrometer from a 50 cm C18 EN PepMap column. Three biological replicates for each cell line were run. Tandem mass spectra were searched against the human subset of the UniProt database using Mascot and peptide identifications were filtered through the Percolator algorithm to achieve a global 1% false discovery rate (FDR). Identifications were imported back into Progenesis QI and mapped onto MS1 peak areas. Peak areas were normalised to total ion intensity for all identified peptides. Relative protein quantification was performed using relative peak areas of non-conflicting peptides. Proteins were accepted for analysis provided they were detected with ≥ 2 peptides and ≥ 1 unique-peptide in at least one sample. Fold changes and p-values for differential abundance were calculated in Progenesis QI by ANOVA.

### Lipidomics analysis of Y201 and Y202 EVs

Lipids were extracted from Y201 and Y202 EVs as previously described [37]. Briefly, 50 µl of the EV suspension was mixed with 3 µl of SPLASH Lipidomix standard, then 997.5 µl of MeOH: CHCl_3_ (2:1, v/v) and 208 µl 0.005 N HCl were added and samples were vortexed followed by the addition of 332 µl of CHCl_3_ and 332 µl of ddH_2_O. The samples were centrifuged at 3600*g* for 10 min at 4 °C, the organic phase was collected and dried in a GeneVac for 30 min. The lyophilised samples were resuspended in 80 µl of injection solvent, ACN: IPA (70:30) and the lipid composition was assessed using liquid chromatography with tandem mass spectrometry (LC-MS/MS). 2 µl of the sample was injected for positive or negative acquisition mode using the Orbitrap Fusion Tribrid mass spectrometer (ThermoFisher Scientific, UK). The lipidomic data were analysed using the online tool MetaboAnalyst 5.0. For the lipidomics dataset vendor .raw files, derived .mzML files, and experimental metadata including lipid annotations were deposited to MassIVE dataset MSV000097745.

### Analysis of Y201 and Y202 EV MicroRNAs (miRNAs)

RNA was extracted using the Total Exosome RNA and Protein Isolation kit (Invitrogen, Cat: 4478545). Samples were thawed and diluted with 1X PBS to 200 µl total volume in an RNAse-free tube. 200 µl of pre-warmed denaturing solution was then added and mixed before incubating samples on ice for 5 min. 400 µl of Acid-Phenol: Chloroform was added to each sample and they were vortexed for 60 s before centrifuging at 13,000*g* for 5 min at room temperature. The aqueous (upper) phase was then transferred to a fresh RNAse-free tube and the volume recovered was recorded and then used in purification. The elution solution was pre-heated to 95 °C and 100% ethanol left at room temperature. The aqueous phase was diluted with 1.25 volumes of 100% ethanol and mixed. The aqueous-phase/ethanol mix was loaded onto a filter cartridge in a fresh tube and centrifuged at 10,000*g* for 15 s. The flow-through was discarded and the cartridge was then washed by centrifuging with 700 µl of miRNA wash solution 1 at 10,000*g* for 15 s. Flow-through was discarded and then 2 washes with 500 µl of miRNA wash solution 2/3 were performed at the same settings. The cartridge was dried with a spin of 10,000*g* for 1 min. The cartridge was removed and placed into a fresh collection tube and the RNA was eluted using 50 µl of pre-heated elution solution and centrifuging at 10,000*g* for 30 s. The eluate was passed through the cartridge a second time to improve yield. The samples were concentrated using Amicon Ultra 0.5 ml centrifugal filters (Sigma-Aldrich, Cat: UFC500308) by first topping up to 400 μL with RNAse-free water, then centrifuging at 14,000*g* for 88 min before inverting and collecting RNA in a fresh tube at 8000*g* for 2 min. Total RNA was then quantified on a Bioanalyzer 2100 (Applied biosystems) using a Pico chip.

5–3 µl of RNA was used in the NanoString miRNA ligation reaction. NanoString was performed following the manufacturer’s miRNA sample preparation protocol using the nCounter Human v3 miRNA expression assay codeset (NanoString). miRNA counts were normalised using miRNA spike-ins for other species following the NanoString procedure. miRNAs with less than 20 counts ≥ 1 sample were filtered out and comparisons of EV miRNAs were performed in R Studio. The TargetScan database was used to identify predicted targets of miRNAs. Targets were included in analyses if satisfying a threshold cut-off of a context score < − 0.6 (the more negative a score the more evidence that the gene is a true target of the miRNA) [38].

### Gene ontology term enrichment and clustering

Gene Ontology (GO) enrichment was performed using the ClueGO plugin for the Cytoscape software package [39, 40]. Gene lists were assessed for enrichment against the Biological Process and Molecular function GO genesets with Benjamini–Hochberg FDR corrected p-values. Redundancy of GO terms was reduced by using the GO-fusion setting in ClueGO before automated clustering of significant GO terms (FDR-corrected *p* < 0.05). Cluster diagrams were generated from ClueGO results using the AutoAnnotate plugin to facilitate organisation and labelling of like-terms and to generate titles for clusters based upon common words [41].

### KEGG pathway enrichment

Lists of significantly more abundant proteins were analysed for pathway enrichment against the curated Kyoto Encyclopedia of Genes and Genomes (KEGG) database using the Molecular Signatures Database website on version 7.2 [42–44] and results filtered to exclude terms with FDR corrected p-values (q) of > 0.05. To minimise the effect of confounding and relatively uninformative terms, a filter excluded gene-sets containing more than 500 genes. Where p-values for enriched pathways were the same, samples were ordered by the MSigDB k/K ratio where k = the number of proteins identified in the geneset and K = the total number of proteins in that set.

### CFSE-EV labelling

Y201 and Y202 EVs were labelled using carboxyfluorescein diacetate succinimidyl-ester (CFSE, Invitrogen, Cat: C34554) by incubating with 20 µM CFSE for 120 min at 37 °C in a final volume of 300 µl PBS. Labelled EVs were pooled by centrifugation at 100,000*g* for 90 min and resuspended in PBS to remove the unbound CFSE dye. Labelled EVs were transferred to a protein LoBind Eppendorf tube for storage.

### Flow cytometric analysis of CFSE labelled EVs

The Cytoflex S system was used with the violet filter being set up as the primary filter to aid in the detection of small particles. PBS was used to calibrate background noise using the violet side scatter channel, as previously recommended [45]. The acquisition settings for the Forward Side Scatter (FSC) were set at 50, FITC at 371 and Violet Side Scatter (VSSC) at 50. Gates to distinguish EVs from the background were set up using a density plot (FSC-H vs. VSSC-H), and the primary threshold of the machine was adjusted to 600 to minimise the detection of buffer noise on the EV gate. The flow rate of the sample was set at 10 µl/min, and events were recorded after allowing the sample to run for the first 60s to ensure a constant flow of the sample. 10,000 EVs were recorded per sample, and the median fluorescence intensity of the EVs was normalised against Y201 EVs. PBS washes were performed between each sample to ensure the removal of all EVs, avoiding cross-contamination of the sample readings.

### Analysis of EV uptake by flow cytometry

Y202 and Y201 MSCs were trypsinised, counted, transferred to LoBind protein tubes (100,000 cells/tube) and treated with Y201 and Y202 CFSE-labelled EVs and unlabelled-EVs at a 10X concentration for 1 h, 2 h, 4 h, 6 h, 8 h and 10 h.

For the RGD-blocking experiments, cells were treated with 200 µΜ Gly-Arg-Gly-Asp-Ser-Pro (GRGDSP) or Gly-Arg-Ala-Asp-Ser-Pro (GRADSP) control peptides (Sigma-Aldrich, Cat: SCP0157, SCP0156) for 6 h before peptide removal by pelleting the cells and resuspending them in fresh DMEM medium supplemented with 10% FBS. CFSE-labelled EVs and unlabelled-EVs at a 10X concentration were exposed to cells for 4 h. Cells were centrifuged at 300*g* for 5 min at 4 °C and washed twice with ice-cold PBS. Cells were resuspended in 200 µl flow buffer (1% FBS in PBS) and EV uptake was determined by flow cytometry using the LX375 CytoFlex. The gains were adjusted as follows: (i) Forward Scatter to 20, (ii) Side Scatter to 40 and (iii) Fluorescein isothiocyanate to detect the CFSE fluorescence at 40.

### Analysis of EV uptake by confocal microscopy

Y202 and Y201 cells were seeded on glass coverslips at a 5000 cells/cm^2^ density and left to adhere overnight. Y201 and Y202 CFSE-labelled EVs or unlabelled EVs were introduced to Y202 and Y201 cells respectively at a 10X concentration for 4 h. Cells were fixed for 10 mins using 3% paraformaldehyde (Thermo Scientific, Cat: 28906) followed by 3 × 5 mins washes with 1 mg/ml NaBH_4_ in PBS to quench free aldehyde groups followed by three washes with PBS. Cells were stained with Phalloidin CruzFluor 594 Conjugate (Santa Cruz, Cat: 363795), for 90 mins in 1% BSA/PBS to visualise actin and mounted with 4′,6-diamidino-2-phenylindole (DAPI, Sigma-Aldrich, Cat: DUO82040). Cells were imaged using a ZEISS LSM 980 laser-scanning confocal microscope (Carl Zeiss, Germany). Solid state lasers of 405 nm (blue), 488 nm (green), and 561 nm (red) excitation wavelengths were used to excite fluorescence with emissions collected at, 415–478 nm, 491–553 nm, and 561 nm, respectively.

### CyQuant proliferation assay

Cell proliferation following EV treatment was determined using the CyQuant assay according to the manufacturer’s protocol. Briefly, Y202 and Y201 MSCs were seeded in a 96-well plate at a 9000 cells/cm^2^ and left to adhere overnight. Y202 MSCs were treated with Y201 EVs and Y201 MSCs were treated with Y202 EVs at 1X, 5X and 10X concentrations. The treatments were calculated using the following equation: (Nt*v*X)/Ni where *Ni* is the number of donor cells, *Nt* number of recipient cells, *v* is the volume of the EV suspension and *X* is the number of the desired treatment. Cells were incubated in the working CyQuant solution for 5–10 min. Quantification of cellular DNA per well was achieved by shaking the microplates for 2 mins at 300 rpm and measuring the fluorescence at 480/502 nm (excitation/emission) using a CLARIOstar plate reader.

### Analysis of cell growth by ptychography

Y202 and Y201 MSCs were seeded in a 24-well plate at 4,500 cells/cm^2^ and left to adhere overnight. Y202 cells were treated with Y201 EVs and Y201 cells were treated with Y202 EVs at 10X concentration. The microplates were loaded onto the LiveCyte microscope and cell proliferation was monitored for 72 h by ptychographic quantitative phase imaging and analysed using the Phasefocus software v3.5.

Primary articular chondrocytes (ACs) were seeded in 24-well plates at a 3000 cell/cm^2^ and left to adhere overnight. Cells were treated with Y201 100K EVs at a 10X and 20X concentrations and cell proliferation was monitored for 72 h using a LiveCyte microscope. For RGD blocking experiments, ACs were treated with 200 µΜ GRGDSP or GRADSP control peptides (Sigma-Aldrich, SCP0157, SCP0156) for 6 h. Cells were washed twice with PBS and Y201 EVs were added (20X concentration). Proliferation rate was monitored for 72 h using the LiveCyte microscope.

### Scratch wound assay

Y202 MSCs were seeded in 24-well plates at 30,000 cells/cm^2^ and left to adhere for 6–8 h in DMEM medium supplemented with 10% FBS, 1% P/S. Cells were washed with PBS and serum-starved overnight. A 10 µl pipette tip was used to create a scratch in the confluent monolayer of cells and the cells were washed with PBS to remove dead cells and debris. Y202 MSCs were treated with Y201 EVs (10X concentration) and wound closure monitored over 24 h by LiveCyte microscopy. Single cell analysis was performed using the MTrackJ plugin in ImageJ and measurements were imported into the chemotaxis tool to calculate the cell velocity, total track length, Euclidean distance, directness and forward migration index.

### Chondrogenic differentiation assay

Primary MSCs were isolated from femoral head donations following ethical approval (LREC 07/Q1105/9). The MSCs were exposed to Y201 or Y202 EVs (10X concentration) for 6 h. Then, micromass pellets were formed at a density of 2.35 × 10^5^ cells per pellet in 100 µl growth media by centrifuging at 300*g* for 5 mins and then incubating overnight at 37 °C.

Pellets were detached from the tube and medium added up to 1.2 ml. Negative controls received basal medium comprising DMEM containing sodium pyruvate and L-glutamine, supplemented with 1% Insulin-Transferrin-Selenium (ITS) + 3 (Sigma-Aldrich, Cat: I2771), 40 µg/ml l-Proline (Life Technologies, Cat: P6698), and 1% non-essential amino acids (Sigma-Aldrich, Cat: TMS-001-C). Chondrogenic medium was composed of basal medium with the addition of 0.1 µM dexamethasone (Sigma-Aldrich, Cat: D2915), 50 µg/ml l-Ascorbic Acid (Sigma-Aldrich, Cat: A4544) and 10 ng/ml TGF-ß1 (Peprotech, Cat: 100-1). Tubes were incubated at 37 °C and medium changed every 3–4 days. Test conditions were treated once per week with 10X EVs per pellet. At day 7, pellets were removed and rinsed twice with 1 ml PBS then fixed for 10 min with cold paraformaldehyde. Pellets were again washed twice with PBS and paraffin wax embedded within 48 h of fixation using a Leica tissue processor. Pellets were then sectioned at 6 μm thickness. To ensure consistency, pellets were sectioned to a depth of 48 microns and then 8 further sections of 6 μm thickness were collected for testing. Sections were cleared and rehydrated prior to staining with 0.02% Fast Green for 5 mins and 0.1% Safranin O for 15 mins. Sections were dehydrated and mounted using DPX mounting medium prior to imaging with Z-stack imaging on an Axio Scan.Z1 slide scanner.

### T cell activation assay

1.0 × 10^5^ primary human peripheral blood-derived CD4+ T cells (Stem Cell Technologies) were grown in RPMI-1640 with 10% FBS 1% P/S (Gibco, Cat: 10363083) and were pre-treated for 6 h with Y201 or Y202 EVs (20X concentration). For positive controls, 1.0 × 10^4^ MSCs were seeded into a 96-well U-bottomed plate and cultured for 24 h prior to addition of 10X peripheral blood-derived CD4+ T cells. Continual proliferative capacity was assessed as a measure of T cell proliferation. CD4+ T cells were stained for 10 min at 37 °C using 1 µM VPD450 Violet proliferation dye (eBioscience, Inc., Cat: 65-0863-14). T cells were activated using anti-CD3ε/CD28 Dynabeads (Gibco, Cat: 10310614) at a bead-to-cell ratio of 1:1 then seeded at a density of 1.0 × 10^5^ cells/well (ratio 10:1) in 200 µl RPMI-1640 with 10% FBS, 0.05 µg/ml IL-2 (Peprotech, Inc, Cat: 200-02). T cells seeded alone (no treatment) or seeded onto 1.0 × 10^4^ Y201 or Y202 MSCs were applied as negative and positive controls respectively and all conditions were tested with and without activation. Plates were cultured for 6 days at 37 °C prior to removal of Dynabeads with the DynaMag-2 as per manufacturer’s recommendations. T cell proliferation was assessed with flow cytometry, with reduction in signal intensity visualised for peaks using FCS Express 7.0 proliferation analysis. Proliferation was assessed through VPD450 dilution (diminished staining intensity) described through a proliferative index (PI) calculated from the fluorescence intensity at each cell division as described previously [33]. Proliferative cycles undertaken were calculated on 50% fluorescence intensity reduction peaks, measuring from fluorescence intensity at Day 0 to the final division detected.

For assessment of T helper differentiation, T cells were activated and cultured with Y201 and Y202 EVs or with MSC monolayers, as described above excluding VPD450 staining. Following 6 days of culture, T cells were re-stimulated using a combination of phorbol 12-myristate 13-acetate (PMA) (50 ng/ml) (Sigma-Aldrich, Cat: P8139) and Ionomycin (1 µg/ml) (Invitrogen, Cat: I24222) and intracellular cytokines retained using transport inhibitor cocktail with 10 µg/ml brefeldin A and 2µM Monensin (Invitrogen, Cat: 00-4980-93). T cells were cultured for 4 h at 37 °C then stained for surface marker CD4 (FITC) (Life Technologies, Cat 11-0049-42) prior to fixation and permeabilisation for intracellular staining of anti-human IFN-γ (Th1) (PE), IL-4 (Th2) (APC) or IL17a (Th17) (PE-Cy7) (Life Technologies, Cat: 12-7319-82, 17-7049-42, 25-7179-42); or CD4 and CD25 (Life Technologies, Cat: 17-0257-42) then fixation/permeabilisation and staining for nuclear protein FOXP3 for regulatory T cells (Life Technologies, Cat: 12-4776-42). All cells were measured using the Cytoflex LX flow cytometer and analysed with FCS Express 7.0. Comparisons were drawn for percentage of T helper differentiation within the CD4+ cell population and signal intensity (Median) for each antibody tested.

### In vivo assessment of immunomodulatory capacity in a murine peritonitis model

All in vivo studies are in line with the Animal Research: Reporting of In Vivo Experiments (ARRIVE) guidelines 2.0 (Supplementary material 1). An in vivo peritonitis model was used in C57BL/6J mice aged 8–10 weeks with zymosan and schistosome egg irritant for induction of inflammation. These experiments were carried out in accordance with the Animals and Scientific Procedures Act 1986, under UK Home Office Licence (project licence number PPL PFB579996 approved by the University of York Animal Welfare and Ethics Review Board). Animals were randomly allocated to the experimental groups and treatment administration was blinded. At day 0, mice were administered with an intraperitoneal infusion of 1 mg of zymosan A (Merck) or 5000 schistosome eggs in 200 µl of PBS. Immediately following administration of irritant, test condition mice were administered an intraperitoneal infusion of EVs isolated from serum-free conditioned media collected over 24 h from either 4.0 × 10^7^ of Y201 MSCs in 100 µl of PBS to treat zymosan-induced inflammation or 2.0 × 10^7^ of Y201 MSCs in 100 µl of PBS to treat schistosome eggs-induced inflammation; negative control mice were given PBS vehicle only (*n* = 3 per condition, *n* = 12 per experiment, *n* = 24 total).

After 24 h, mice were euthanised using CO_2_ overdose and cervical dislocation. Intraperitoneal injection of 4 ml of ice cold RPMI-1640 was administered as peritoneal lavage. The process was repeated with a second 4 ml RPMI-1640 wash and solutions pooled to form the peritoneal exudate cells (PEC). For each animal tested prior to flow cytometric analysis, red blood cells were lysed from the PEC using Red Cell Lysis buffer (Merck) and a cell count performed. PEC samples were initially stained for Ly6C (APC), F4/80 (PE-Cy7), CD45 (PerCP-Cy5.5) (Biolegend, Cat: 128016, 123114, 103132) and Ly6G (FITC), CD11b (BUV395) and SiglecF (BV421) (BD, Cat: 551460, 563553, 562681). PEC samples were then stained for TCRb (AF488), CD3 (APC-Cy7), CD4 (PerCP-Cy5.5), CD62L (APC) and CD44 (PE) (BioLegend, Cat: 109215, 100222, 100540, 104412, 103008). For all tests, Zombie Aqua (BioLegend, Cat: 423101) was used to exclude dead cells.

### Antigen-induced arthritis model

The antigen-induced arthritis (AIA) model of inflammatory arthritis was used in male C57Bl/6 mice aged 7–8 weeks as previously described [46] with all procedures performed under Home Office project licence P0F90DE46. Animals were randomly allocated to the experimental groups. Treatments were administered as intra-articular injections of EV suspensions in 15 µl of PBS corresponding to the number of EVs produced by ~ 5.0 × 10^6^ cells, or PBS only controls, with *n* = 4 for each condition. Mice were injected 1 day post arthritis induction using 0.5 ml monoject (29G) insulin syringes (BD Micro-Fine, Franklyn Lakes, NJ, USA) through the patellar ligament into the knee joint. Where anaesthesia was required, it was administered and maintained for the duration of the procedure. This was assessed via the complete loss of the pedal reflex. We used inhalant anaesthesia using isoflurane for the intra-articular injections. To avoid hypothermia after anaesthesia, we used heating pads to maintain body temperature. Swelling was determined by measuring knee joint diameter at 1-, 2- and 3-days post-injection using a digital micrometre (Kroeplin GmbH, Schlüchtern, Germany). Peak swelling caused by inflammation was observed at 24 h post-induction and diameters are reported as reduction from peak swelling. Animals were sacrificed for histological analysis at day 3 post arthritis induction. Joints were fixed in 10% neutral buffered formal saline and decalcified in formic acid for 4 days at 4 °C before embedding in paraffin wax. Tissue sections (5 μm) were stained with haematoxylin and eosin (H&E, Merck Life Science UK Ltd., Cat: HHS32, HT110132) and mounted in Hydromount (Scientific Laboratory Supplies Ltd., Cat: Nat1324). H&E sections were scored for synovial hyperplasia (0 = normal to 3 = severe), cellular exudate (0 = normal to 3 = severe) and synovial infiltrate (0 = normal to 5 = severe) by two independent observers blinded to experimental groups. The scores were summated to produce a mean arthritis index.

### Statistical analysis

The statistical analyses for all experiments were performed using GraphPad Prism v9.0.2. The statistical significance between treatment and control (DMEM) was assessed by *t*-test, One-Way ANOVA or Two-Way ANOVA and Bonferroni or Tukey corrections. Error bars show Standard Error of the Mean (SEM) and asterisks represent the following P values, *p < 0.05, **p < 0.01, ***p < 0.001, ****p < 0.0001.

## Results

### Characterisation of MSC EV subtypes

**Fig. 1:**
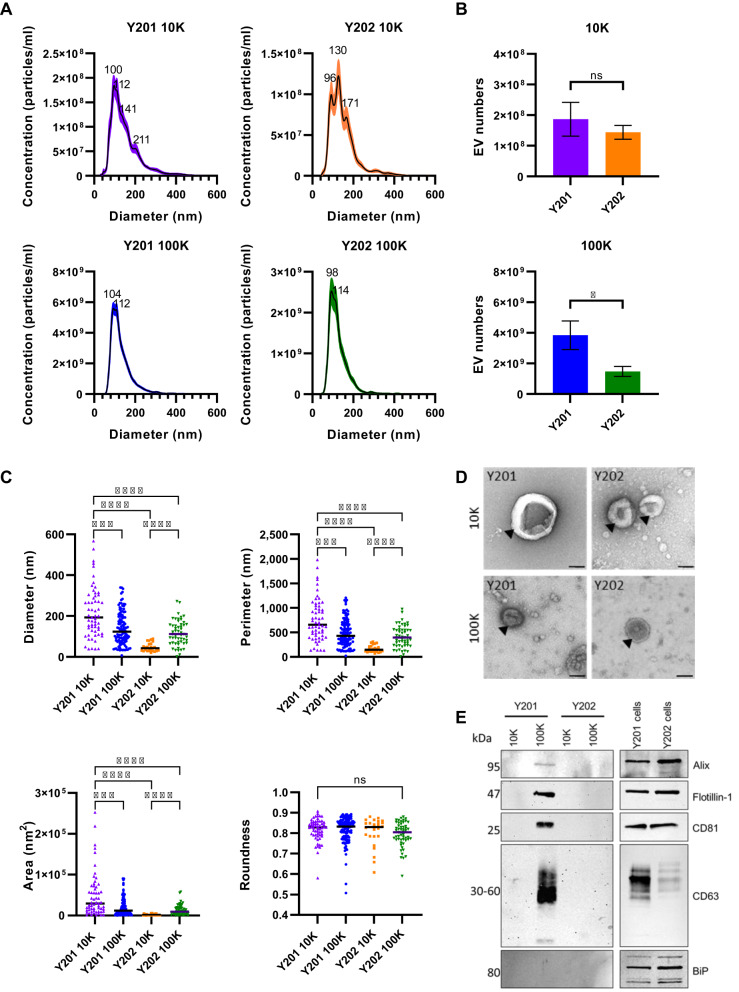
Characterisation of EVs isolated from MSC subtypes. **A** Nanoparticle tracking analysis was used to determine sizes of the 10K and 100K EV fractions from Y201 and Y202. **B** Quantification of EVs using the LM14 Nanosight 3.4 software. **C** Morphological analysis of TEM images was performed using TEM ExosomeAnalyzer to determine diameter, perimeter, area and roundness. **D** Negatively stained EV samples imaged using TEM, Scale bar: 100 nm. **E** Western blot analysis of common EV markers (Alix, Flotillin-1, CD81, CD63) and negative control (BiP). Tukey’s (One-Way ANOVA) statistical test *p < 0.05,,***p < 0.001, ****p < 0.0001. Blots have been cropped for clarity and conciseness. Full-length blots are presented in Supplementary Figure S1a

The size and yield of EVs derived from Y201 and Y202 MSCs were determined by Nanoparticle Tracking Analysis (NTA). We identified three populations in the Y201 10K fraction with peak sizes of 100 ± 18.25 nm, 112 ± 3.8 nm, and 211 ± 12.6 nm. The Y202 10K fraction had three distinct populations with peak sizes of 96 ± 8.6 nm, 130 ± 0.5 nm and 171 ± 4.2 nm (Fig. 1A). The mean size of vesicles isolated from the Y201 and Y202 100K fractions was calculated to be 133.5 nm and 124.8 nm respectively. In the Y201 100K fraction we identified one distinct population with a modal size of 104 ± 12 nm and a second peak at 112 ± 1 nm. The Y202 100K fraction also had one distinct population with a modal size at 99 ± 8 nm and another peak at 114 ± 2.2 nm (Fig. 1A). The mean size of vesicles isolated from the Y201 and Y202 100K fractions was calculated to be 133.5 nm and 124.8 nm respectively. Collecting EVs from non-proliferating cells allowed the estimation of the secreted EVs per million cells. There were no significant differences between Y201 and Y202 10K EV yields, whereas Y201 cells secreted a significantly higher number of EVs isolated in the 100K fraction compared to Y202 cells (Fig. 1B). Both the 10K and 100K fraction of Y201 and Y202 EVs had typical morphological characteristics, as demonstrated by negative staining followed by TEM. The TEM images were analysed with the TEM ExosomeAnalyser software for morphometric quantification of the EVs [47]. In general terms, the Y201 10K EVs were more variable and larger than the other fractions but there were no significant differences between Y201 and Y202 100K EVs in any morphological parameters measured (Fig. 1C, D). TEM analysis showed the Y201 100K fraction had a mean diameter of 138.5 nm whereas the Y202 100K fraction had a mean diameter of 116.3 nm, corresponding closely with the NTA data. Western blotting demonstrated that Flotillin-1, Alix, CD63, CD81 were enriched in the Y201 EV fractions, particularly in 100K, compared to Y202 EV fractions (Fig. 1E and Supplementary material 2: Supplementary Fig. S1). The presence of the endoplasmic reticulum protein, binding immunoglobulin protein (BiP), in the cell lysates and absence in the EV fractions confirmed the purity of the EV suspensions. Considering these data, subsequent work focused on the 100K EV fraction.

### Analysis of Y201 and Y202 EV miRNA cargo

**Fig. 2:**
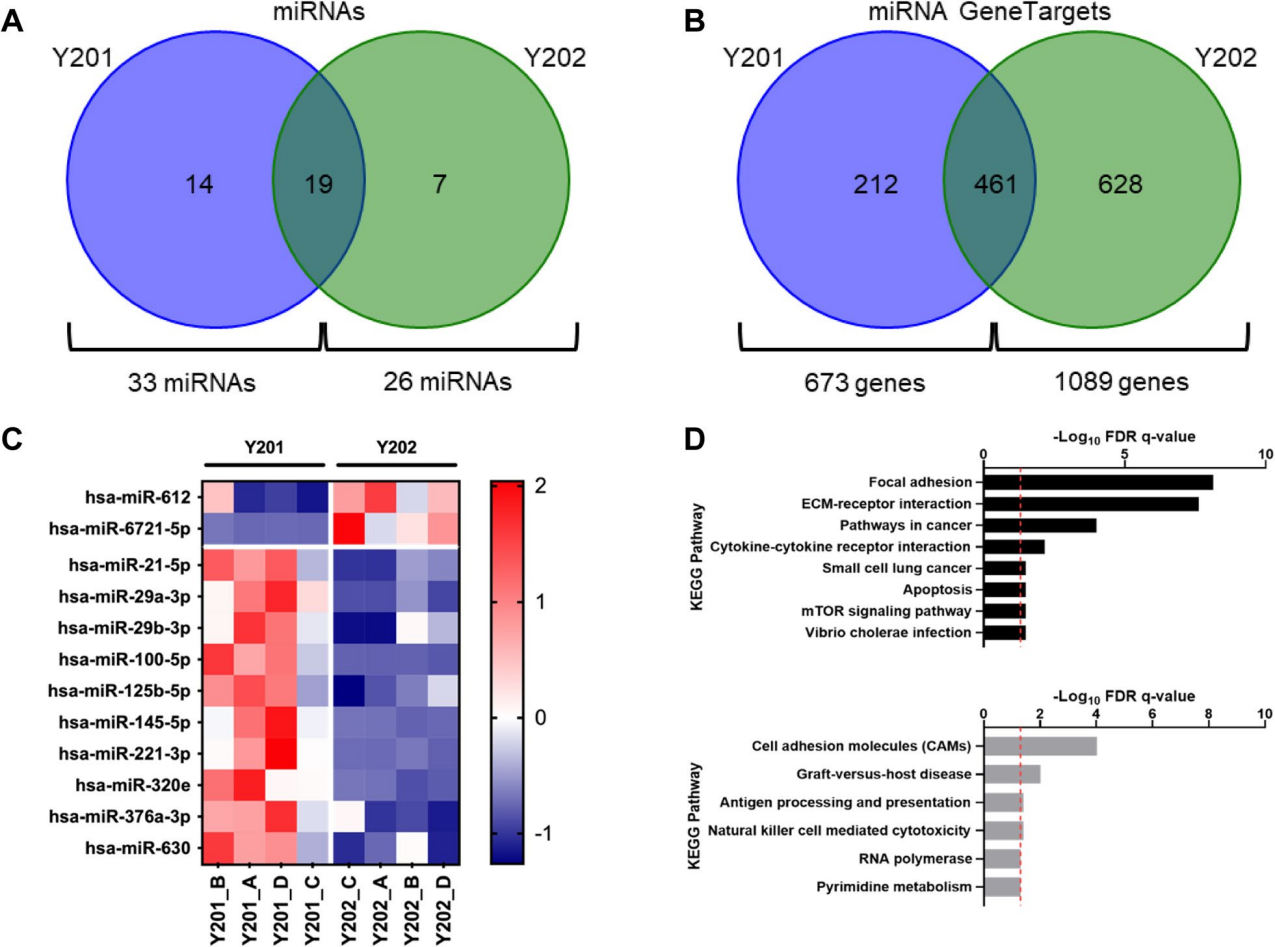
Analysis of Y201 and Y202 EV miRNA cargo. **A** Presence and absence of miRNAs across Y201 and Y202 EVs displayed as Venn diagram. **B** MIENTURNET miRTarBase analysis of genes targeted by miRNAs packed into Y201 and Y202 EVs miRNAs. **C** Clustered heatmap of 12 significantly differently expressed miRNAs identified in EVs after Nanostring miRNA quantification between Y201 and Y202 cells. 10 miRNAs were upregulated in Y201 EVs versus 2 in Y202 EVs, *n* = 4, ANOVA, *p* < 0.05. Colour scale is representative of z-score. **D** KEGG pathway enrichment for predicted targets of miRNAs that were more abundant in Y201 vs. Y202 EVs. Significantly enriched KEGG pathways (FDR corrected q-value < 0.05) for genes that were predicted targets of the 10 miRNAs that were more abundant in Y201 EVs (black, top) and Y202 EVs (grey, bottom). Red line marks q < 0.05. q = FDR corrected p-value

We next analysed the miRNA content of EVs (100K fraction) from Y201 and Y202 cells. miRNA detection was achieved using the Nanostring nCounter using a codeset with 828 miRNA probes; miRNAs with more than 20 copies on average were considered present in the 100K EV fraction. Y201 cells packaged 33 miRNAs, whereas Y202 cells packaged 26 miRNAs, 19 of which are mutual for Y201 and Y202 EV populations (Fig. 2A and Supplementary material 3). These miRNAs were assigned to their experimentally validated target genes on the miRTarBase online database. The 33 Y201 EV-miRNAs target 673 different genes, and the 26 Y202 EV miRNAs target 1089 genes; 461 of these genes are targeted by both Y201 and Y202 EV-miRNA (Fig. 2B). The fold change of the EV miRNAs calculated for all miRNAs that had, on average, more than 20 counts in either Y201 or Y202 EVs are provided in Supplementary material 2: Supplementary Table S1. An unpaired *t-test* with multiple corrections using the absolute miRNA numbers from both Y201 and Y202 EVs was performed. Ten miRNAs were significantly upregulated in Y201 EVs versus Y202 EVs, and two were significantly upregulated in Y202 EVs versus Y201 EVs (Fig. 2C). The largest fold change in Y201 EVs was found in miR-100-5p, whereas in Y202 EVs it was miR-6721-5p. Two of the three miRNAs comprising the highly conserved miR-29 family (miR-29a-3p and miR-29b-3p) were increased in Y201 EVs versus Y202 EVs and of the miRNAs elevated in Y201 EVs, miR-125b-5p had the highest mean miRNA count. We used the TargetScan miRNA prediction algorithm to identify possible targets for the enriched miRNAs in both Y201 and Y202 EVs. The 10 miRNAs elevated in Y201 EVs produced 378 predicted targets while the two miRNAs increased in Y202 EVs targeted a possible 743 mRNAs (total-context++ − <0.6) [38]. The list of potential targets for each miRNA was collectively assessed using the KEGG pathway databases. Enrichment was identified for the “Focal adhesion” and “ECM-receptor interaction” pathways for Y201 EV miRNAs (Fig. 2D). Despite having a greater number of predicted targets, the Y202 EV miRNA targets did not collectively enrich for any GO terms. However, when compared against KEGG pathways, six pathways were enriched at low significance, with the “Cell adhesion molecules’’ pathway most significant (Fig. 2D).

### Analysis of Y201 and Y202 EV lipid composition

LC-MS/MS was used to determine the lipid profile of EVs derived from Y201 and Y202 MSCs. We identified 172 different lipid species, though only 7 had significantly different abundances between the 2 groups with > 2-fold change (*p* < 0.05, t-test). Principal component analysis (PCA) plots showed some separation between Y201 and Y202 EV datasets, though with significant overlap, which was replicated using a hierarchical clustering heatmap, indicating similar lipidomic features across the two EV populations (Supplementary material 2: Supplementary Fig. S2A–C).

Comparable lipid signatures for Y201 and Y202 EVs were also revealed based on the most abundant lipids. EVs derived from both Y201 and Y202 MSCs, had a high abundance of phosphatidylcholines (PCs) and sphingomyelins (SMs), particularly, PC(16:0_18:1), and SM(d18:1/16:0) (Supplementary material 2: Supplementary Tables S2 and S3). We attempted to define those lipids that were significantly different in Y201-derived EVs in comparison to Y202-derived EVs, but few of these were identifiable. It is notable however, that there was a significant enrichment of the lactosylceramide LacCer(d18:1/22:0) in Y201 EVs compared to Y202 EVs (Supplementary material 2: Supplementary Table S4).

### Analysis of Y201 and Y202 EV protein cargo

**Fig. 3:**
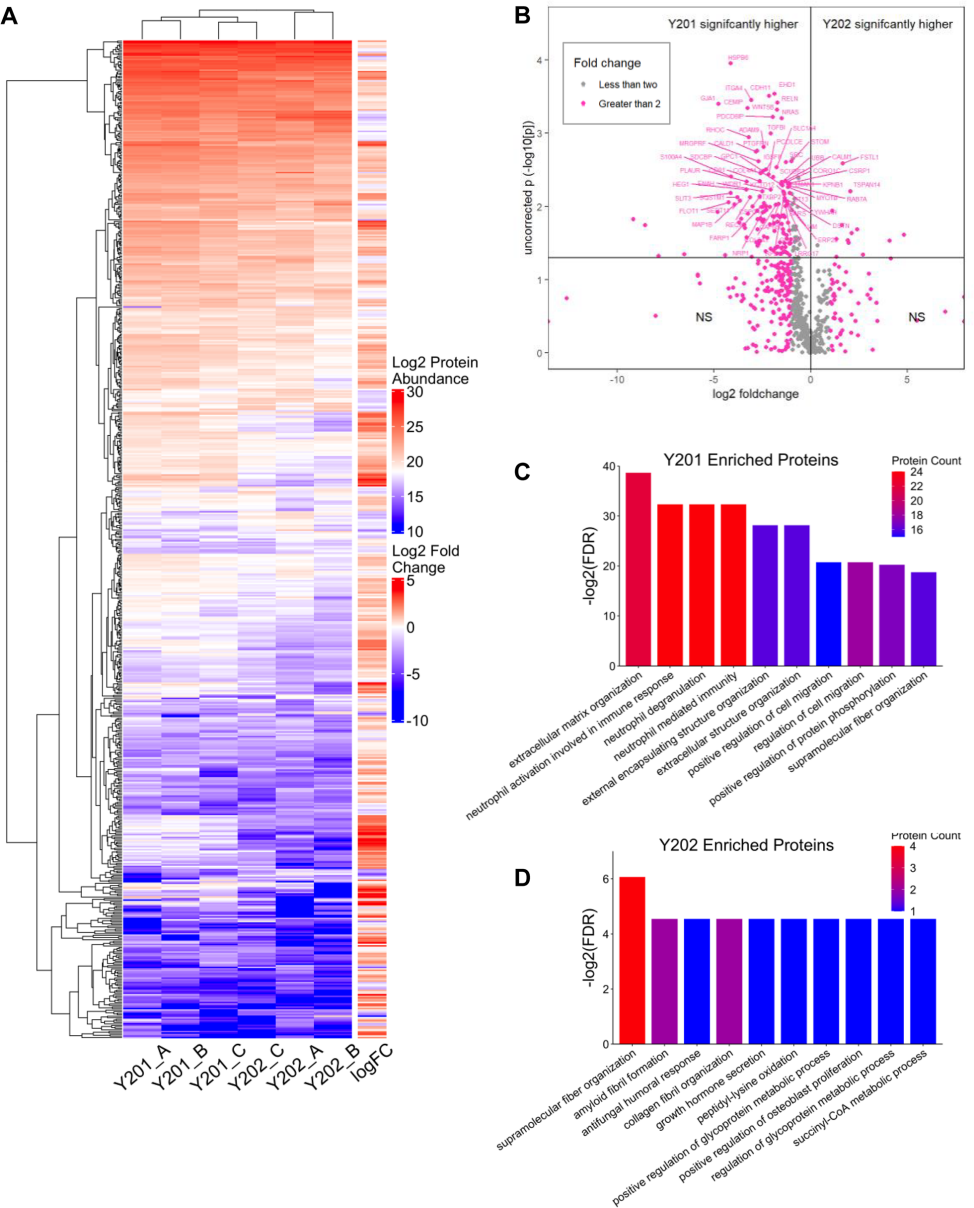
Analysis of Y201 and Y202 EV protein cargo. **A** Hierarchical clustering heatmap of protein abundance for shared proteins among Y201 and Y202 EVs. Higher levels of protein abundance are indicated in shades of red, and lower levels are indicated in shades of blue. **B** Volcano plot of proteins identified in EV isolations from Y201 and Y202 clonal lines. Volcano plot of fold change for the 663 proteins identified from LC-MS/MS analysis of EVs isolated from Y201 and Y202. Proteins with a fold change > 2 between cell lines are shown in pink with fold changes < 2 in grey. The two upper quadrants contain proteins that were significantly different between Y201 and Y202 as determined by ANOVA (*p* < 0.05). **C** Enrichment analysis against Biological Process Gene Ontology geneset for proteins enriched in Y201 EVs and **D** those enriched in Y202 EVs. Shown are the top 10 most significant processes as measured by −log10 of the adjusted p-value (FDR) and total number of proteins in the dataset which match each term. Higher protein count indicated in red and lower protein count indicated in blue

Next, EVs (100K) isolated from Y201 and Y202 cells were analysed by LC-MS/MS to determine protein content. Hierarchical clustering analysis was performed to compare overall abundances of shared proteins between Y201 and Y202 EVs (Fig. 3A). Although both EV subtypes are relatively homogenous in their composition of proteins, there are variations in protein abundances. From a total of 663 proteins identified across all samples, there was a significantly increased abundance of 162 proteins in Y201 EVs compared to 14 proteins that were significantly more abundant in Y202 EVs (ANOVA, *p* < 0.05) (Fig. 3B and Supplementary material 3). To investigate differential enrichment further, the top and bottom 25 most abundant EV proteins were visualised in a heatmap (Supplementary material 2: Supplementary Fig. S3), demonstrating that most of the proteins enriched in Y201 EVs have a larger fold change compared to those in Y202 EVs. To explore potential functional differences between the most abundant Y201 or Y202 EV proteins, enrichment analysis was conducted against the GO Biological Process geneset. In terms of Y201 enriched EV proteins, extracellular matrix (ECM) organisation and terms relating to neutrophil mediated immunity were among the most upregulated processes (Fig. 3C). Statistically more abundant proteins in Y202 EVs were enriched for pathways including supramolecular fibre organisation, amyloid fibril formation, and anti-fungal humoral response (Fig. 3D). Notably however, due to the low number of proteins significantly enriched in in Y202 EVs versus Y201 EVs (14 and 162 respectively),

the only notable enriched processes were those with a low number of total associated proteins (compare Fig. 3C and D).

**Fig. 4:**
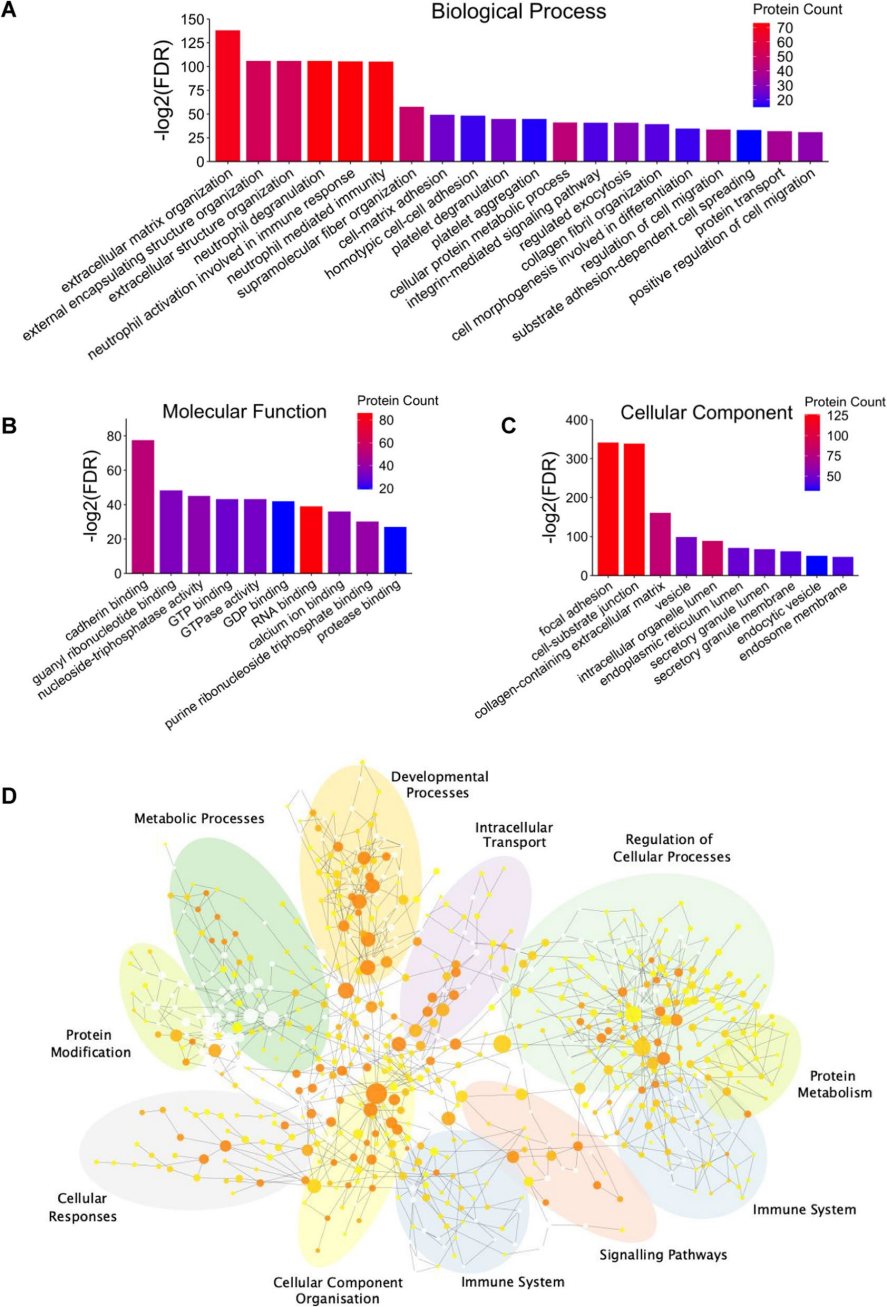
Functional enrichment analysis of the Y201 EV proteome. Enrichment analysis against **A** Biological Process, **B**) Molecular unction and **C** Cellular Component Gene Ontology (GO) terms for the Y201 proteome. Only the most significant terms are shown, as determined by -log10 adjusted p-value (FDR) and number of proteins associated with each term. Higher protein count indicated in red and lower protein count indicated in blue. **D** BiNGO network of significant GO Biological Process terms for the Y201 proteome, annotated to cluster common terms by similar processes using Biorender. Darker shades of orange correspond to higher significance (p-value threshold < 0.05)

To provide further insight into the nature of the enhanced Y201 EVome, GO analysis against Biological Process, Molecular Function, and Cellular Component genesets was carried out. In terms of Biological Process, the Y201 EVome is predominantly enriched in organisation and structure of the extracellular matrix, immunomodulatory activity mediated through neutrophils, and various adhesion and migration processes (Fig. 4A). The Molecular Functions of these proteins largely pertain to the binding of cadherin and various nucleic acids including GDP, nucleic triphosphates, and RNAs (Fig. 4B). Enrichment against the Cellular Components geneset revealed that these proteins are significantly present in focal adhesions and cell-substrate junctions, to a relatively substantial degree. (Fig. 4C). To gain a broader perspective of potential Y201 EV functions in vivo, the Y201 EVome was assessed for enrichment against the GO Biological Process geneset in the BiNGO plugin for Cytoscape, which formulates clustered networks of significantly enriched processes (Fig. 4D). Sizeable clusters formed around similar significant processes relating to the immune system, regulation of cellular processes, developmental processes, and cellular component organisation. In comparison, running the same analysis in BiNGO for the Y202 EVome identified some overlap with the Y201 EVome, with clusters around immune response and developmental processes as well as cell migration/transport, protein metabolism and response to stress (Supplementary material 2: Supplementary Fig. S4A).

Considering the most abundant Y201 EV proteins were associated with ECM organisation, we attempted to model the extravesicular corona by constructing a protein-protein interaction (PPI) network in STRING (Supplementary material 2: Supplementary Fig. S4B). We identified a large number of PPIs between ECM component and cognate vesicle membrane integrins and tetraspanins. Fibronectin (FN1) and milk fat globule epidermal growth factor 8 (MFG-E8) were amongst the most abundant presumptive Y201 EV coronal proteins, which we confirmed by western blot analysis relative to Y202 EVs (Supplementary material 2: Supplementary Fig. S4C). Collectively, these data demonstrated that EVs released by MSC subtypes isolated from the same donor, have broadly similar morphological characteristics but differ significantly in terms of yield, EV marker abundance and cargo.

### Uptake of EVs by different MSC subtypes

To determine EV uptake by recipient Y202 and Y201 cells, EVs from each cell line were fluorescently-labelled using CFSE and internalised signal measured by flow cytometry and confocal microscopy. The CFSE intensity was normalised to Y201 100K EVs, and it was demonstrated that Y202 100K EVs have significantly lower fluorescent intensity by approximately 20%. All the median values obtained using Y202 EVs were normalised against Y201 EVs for the subsequent experiments (Supplementary material 2: Supplementary Fig. S5). Y201 and Y202 cells were treated with Y202 and Y201 CFSE-labelled EVs respectively, and flow cytometry was used to quantify EV uptake by the cells. A gating strategy using the Cytoflex LX375 was set up using the following parameters. An FSC against SSC plot was created to discriminate between cells and debris in suspension. After gating out the debris, single cells were distinguished by plotting FSC-H against FSC-A. Cells that were not treated with stained EVs were used to identify the autofluorescence of the cells by plotting SSC against the CFSE signal. Finally, cells treated with CFSE-EVs were used to measure the successful detection of EV uptake. An approximate signal of 3000 arbitrary units (AU) on the CFSE parameter was considered the background signal of both Y201 and Y202 cells (Supplementary material 2: Supplementary Fig. S6).

**Fig. 5:**
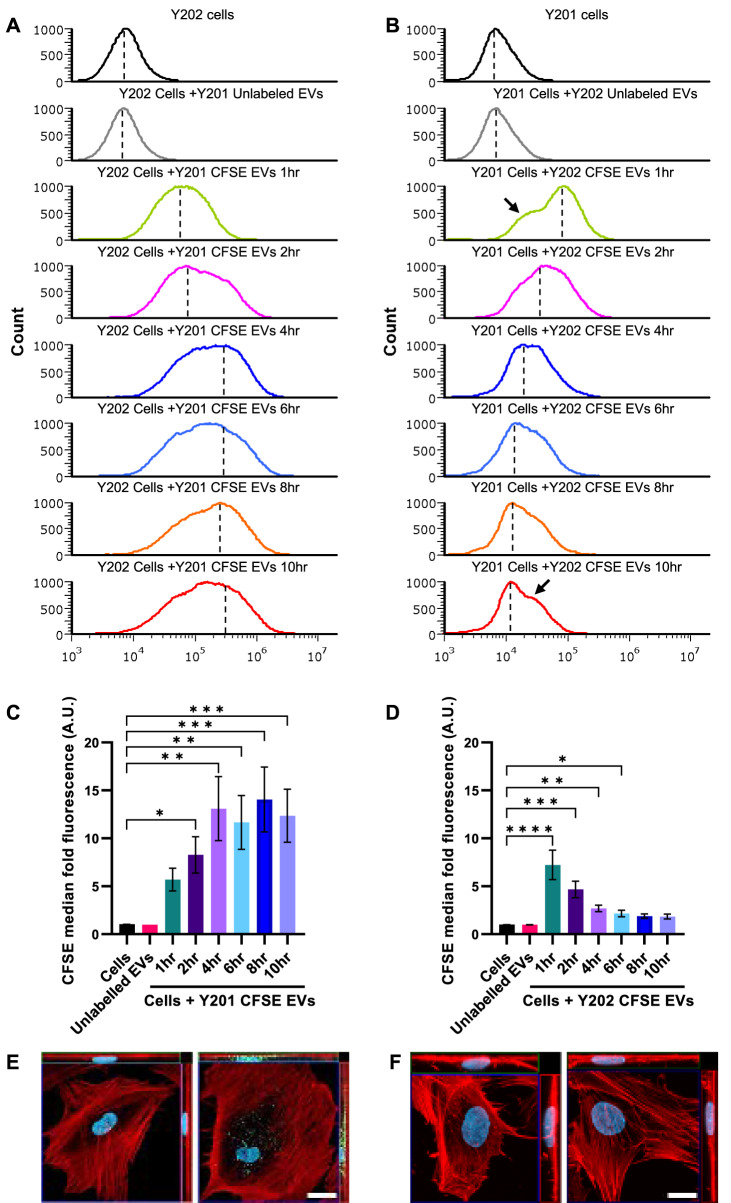
Uptake of Y201 EVs by Y202 cells (**A**, **C**, **E**) and Y202 EVs by Y201 cells (**B**, **D**, **F**). **A**, **B** Y202 and Y201 cells were treated with CFSE-labelled Y201 and Y202 EVs respectively. CFSE signal in the cells was monitored for 10 h and histograms (counts vs. CFSE) signals were normalised to peak values. Dashed lines represent the median values used to quantify CFSE signal levels relative to control (cells), arrows (in part **B**) show the presence of a putative subpopulation of cells, **C**, **D** quantification of the median CFSE intensity relative to control were plotted as bar graphs. *n* = 3, error bars = SEM, Kruskal-Wallis test, *p < 0.05, **p < 0.01, ***p < 0.001, ****p < 0.0001. **E** confocal microscopy orthogonal projections of Y202 cells exposed to CFSE-labelled Y201 EVs for 4 h and **F** Y201 cells exposed to CFSE-labelled Y202 EVs for 4 h. Scale bar: 20 μm, red = rhodamine-phalloidin, blue = DAPI, green = CFSE

Y202 cells exposed to CFSE-labelled Y201 EVs increased in fluorescence over the first 4 h and levels remained elevated for up to 10 h (Fig. 5A, C). Orthogonal projections by confocal microscopy after 4 h of CFSE-labelled Y201 EV exposure, confirmed the fluorescent signal observed was from intracellular EVs in Y202 cells (Fig. 5E). When CFSE-labelled Y202 EVs were added to Y201 cells, only a slight increase in fluorescence was detected after 1 h, with levels decreasing over the remaining time course (Fig. 5B, D). In addition, Y202 CFSE-EVs could not be observed by confocal microscopy in Y201 cells after 4 h (Fig. 5F). These findings demonstrate that different MSC subtypes, Y201 and Y202, and the EVs they produce display distinct uptake kinetics, which is likely to impact biological function.

### Y201 EVs promote proliferation of Y202 cells

**Fig. 6:**
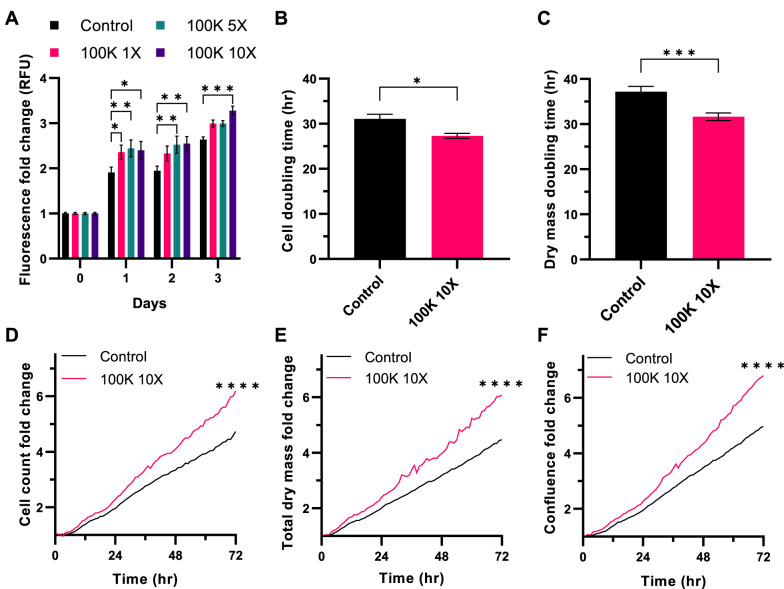
Effect of Y201 EVs on Y202 proliferation. **A** Y202 cells treated with 1X, 5X and 10X concentrations of Y201 EVs and cell number determined over 72 h (CyQuant assay). Livecyte image analysis was used to determine the effect of 10X Y201 EVs on Y202, **B** cell doubling time, **C** dry mass doubling time, **D** cell counts, **E** dry mass, and **F** Y202 confluence. *n* = 3 t-test or Two-Way ANOVA *p < 0.05, ***p < 0.001, ****p < 0.0001

**Fig. 7:**
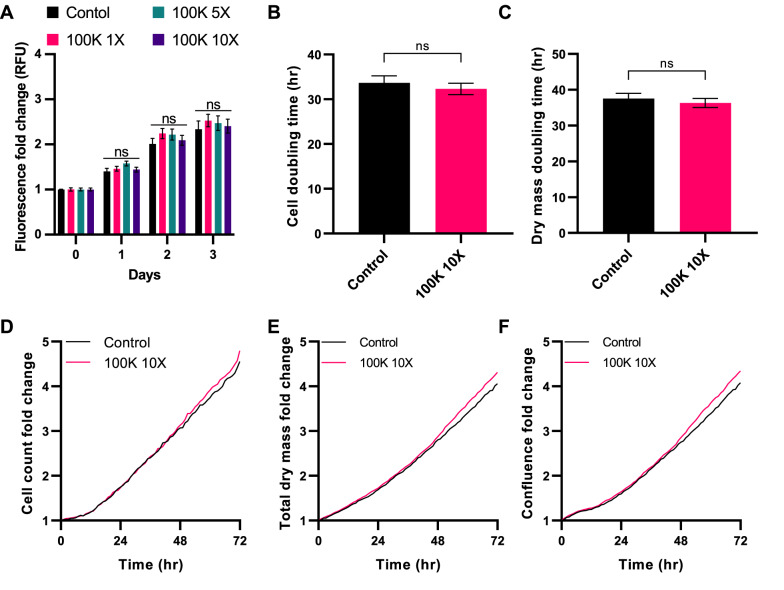
Effect of Y202 EVs on Y201 proliferation. **A** Y201 cells treated with 1X, 5X and 10X concentrations of Y202 EVs and cell number determined over 72 h (CyQuant assay). Livecyte image analysis was used to determine the effect of 10X Y202 EVs on Y201 **B** cell doubling time, **C** dry mass doubling time, **D** cell counts, **E** dry mass, and **F** Y201 confluence. *n* = 3, t-test or Two-Way ANOVA, ns = not significant

To test bioactivity, Y201 EVs were introduced to Y202 cells daily and cell numbers were quantified using a CyQuant assay. The 100K EV fraction from Y201 cells at 1X, 5X and 10X treatments induced a significant increase in DNA content with the 10X concentration maintaining significance 72 h after the initial treatment (Fig. 6A). The proliferative effect of Y201 EVs on Y202 cells was also examined using quantitative live imaging (Livecyte ptychography microscopy) that enables simultaneous measurement of several different parameters associated with proliferation at a single cell level, including: doubling times, dry mass, cell counts and confluency. We also demonstrated that a single treatment with Y201 EVs stimulated growth of Y202 cells with doubling times significantly reduced when measured by cell count or dry mass (Fig. 6B, C) with significant increases in total cell counts, total dry mass and confluency over 72 h compared to untreated controls (Fig. 6D–F). Conversely, EVs derived from Y202 cells had no significant effect on Y201 cells in the same proliferation assays (CyQuant, livecyte doubling times, dry mass, cell counts, confluency) at any time point (Fig. 7). In addition, we found that the introduction of Y201 EVs to Y201 cells had no effect on viable cell numbers when added every 24 h overs a 3-day period, at 1X, 5X and 10X concentrations (data not shown). This observation may the reflect the lack of any additive effect of EV addition to the parent cell against a background of autocrine EV-mediated proliferative effects.

### Y201 EVs promote the migration of Y202 cells

**Fig. 8:**
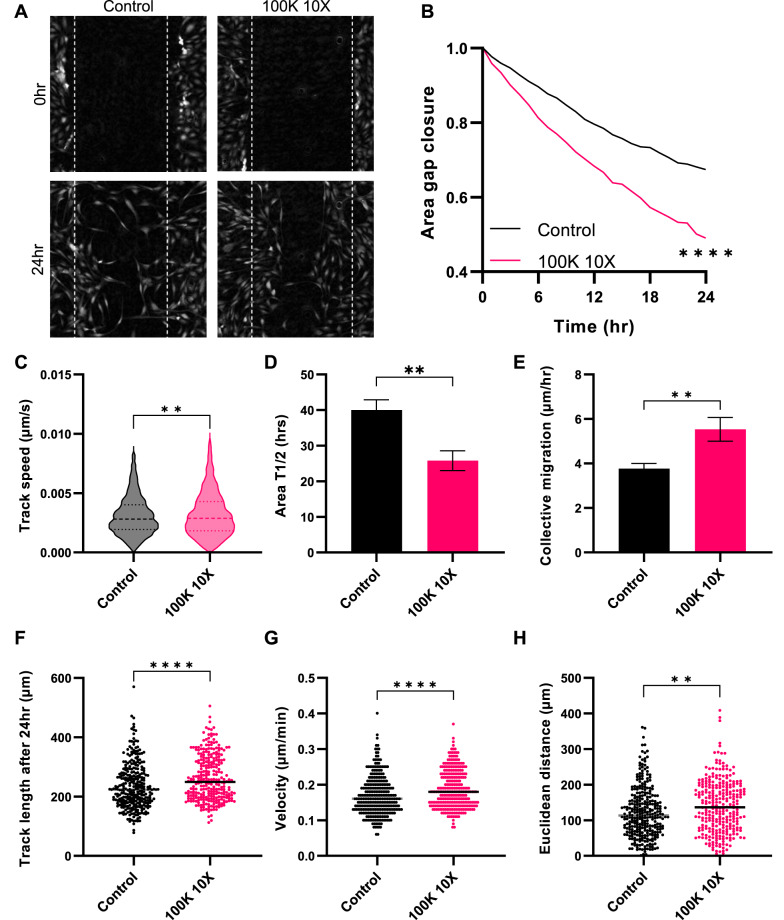
Effect of Y201 EVs on migration of Y202 cells. The scratch wound assay was used to monitor Y202 migration with and without exposure to Y201 EVs (100K 10X) using Livecyte image analysis. **A** Micrographs of untreated control (left) and Y201 EV-treated (right) scratch-wounds at 0 h (top) and 24 h (bottom). Effect of Y201 EVs on **B** gap area, **C** Y202 track speed, **D** area T1/2, **E** Y202 collective migration, **F** Y202 track length, **G** cell velocity and **H** Euclidean distance compared to untreated controls. *n* = 3, t-test or Two-Way ANOVA *p < 0.05, **p < 0.01, ***p < 0.001, ****p < 0.0001

Scratch wound assays were used to test the effect of Y201 EVs on Y202 cell migration using LiveCyte image analysis. We demonstrated that Y201 EV exposure induced a significant increase in wound gap closure compared to untreated controls (Fig. 8A, B). The track speed of cells at the leading edge of the scratch wound was also significantly increased following EV exposure compared to controls (Fig. 8C). The time needed to cover 50% of the gap area was significantly decreased by 9 h and the collective migration of the cells (a measure of how fast the cells on the edge move, assuming that cells in both edges move at the same rate) was increased by approximately 2 μm/h with Y201 EV treatment compared to controls (Fig. 8D, E). Single cell analysis employing the MTrackJ and chemotaxis plugins in ImageJ were also performed. These analyses demonstrated that the track length, velocity and forward migration index of treated cells were significantly increased with Y201 EVs compared to the untreated group (Fig. 8F–H). Y202 EVs had no effect on Y201 migration (Supplementary material 2: Supplementary Fig. S7).

### Effect of Y201 EVs on articular chondrocyte proliferation

**Fig. 9:**
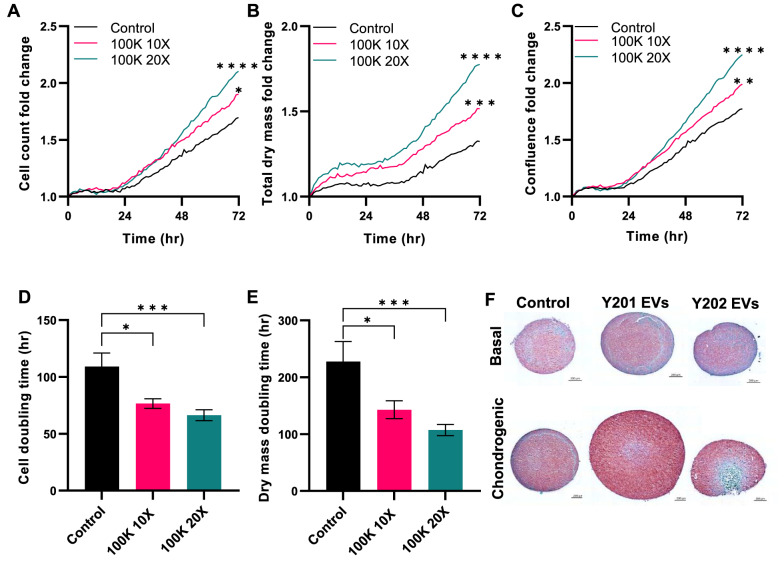
Effect of Y201 EVs on proliferation of primary articular chondrocytes and chondrogenesis. Primary articular chondrocytes were exposed to Y201 100K EVs at 10X and 20X concentrations. Cell proliferation was monitored by LiveCyte imaging over 72 h determining (**A**) cells counts, **B** total dry mass, **C** confluence, **D** cell doubling time and **E** dry mass doubling time. *n* = 3 Two Way-ANOVA followed by Tukey multiple comparison *p < 0.05, **p < 0.01, ***p < 0.001, ****p < 0.0001. **F** Primary donor MSCs as micromass pellets in basal or chondrogenic differentiation conditions for 7 days with or without exposure to Y201 EVs and Y202 EVs. Micromasses were fixed, sectioned and stained with Safranin O. Red staining indicates chondrogenic differentiation. Image shows representative staining in one primary donor

The proliferative effect of Y201 EVs was also examined using primary articular chondrocytes (ACs) isolated from three samples from osteoarthritis patients. The ACs were treated with 10X and 20X concentrations of Y201 EVs and proliferation was monitored for 72 h using ptychographic quantitative phase imaging. ACs treated with Y201 EVs resulted in a significant increase in total cell counts, total dry mass and confluency over 72 h in a dose-dependent manner (Fig. 9A–C). The effect was particularly prominent during the final 24 h of the experiment for the 20X treatment and the final 12 h for the 10X treatment. AC doubling time was reduced by approximately 33 h and by 43 h for 10X and 20X treatments respectively compared to the controls. AC dry mass was reduced by 107 h and 157 h for 10X and 20X treatments respectively compared to the controls (Fig. 9D, E). Y202 EVs had no significant effect on any measure of AC proliferation (Supplementary material 2: Supplementary Fig. S8).

Considering these observations, we evaluated the chondrogenic effect of the Y201 and Y202 EVs. Micromass pellets were formed using primary MSCs, treated with Y201 and Y202 EVs and cultured in basal or chondrogenic differentiation medium for 7 days. Following histological staining with Safranin O, which identifies cartilage proteoglycans, we demonstrated that Y201 EV-treated pellets were significantly larger with more intense Safranin O staining compared to Y202 EVs and untreated controls (Fig. 9F).

### RGD-integrin mediated uptake and function of Y201 EVs

**Fig. 10:**
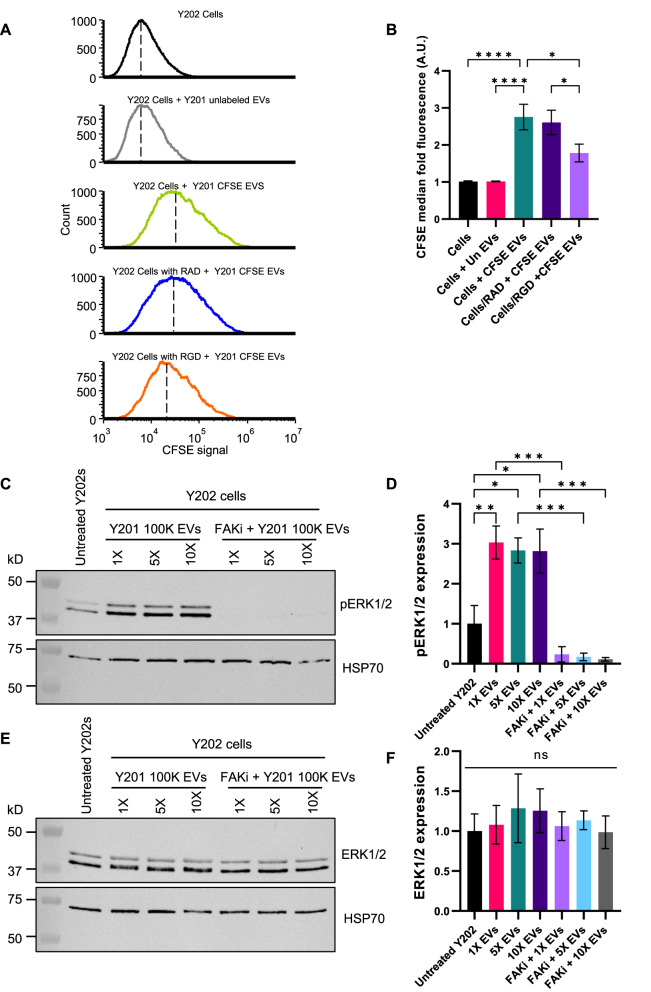
Mechanistic analysis of Y201 EVs. The uptake of CFSE-labelled Y201 EVs cells, with and without RGD blocking peptides (GRGDSP) or RAD control peptides (GRADSP), and effect on phosphorylation of ERK1/2 (pERK1/2) was determined in Y202 cells. **A** Histograms showing CFSE signal in Y202 cells after exposure to CFSE- labelled Y201 EVs for 4 h. Green histogram shows EV uptake in untreated control Y202 cells; blue histogram shows EV uptake in the presence of RAD peptides; orange histogram shows EV uptake in the presence of RGD peptides. **B** Fold change quantification of the CFSE median fluorescence of the Y202 cells, *n* = 6. **C**–**F** Western blot analysis of the effect of Y201 EVs on phosphorylation of ERK1/2 (pERK1/2) (**C** & **D**) and total ERK1/2 (**E** & **F**) in Y202 cells. Y202 cells were treated with Y201 EVs for 30 min at various concentrations (1X, 5X and 10X) in the absence and presence of a focal adhesion kinase inhibitor (FAKi) and western blotting (**C** & **E**) with quantification by densitometry (**D** & **F**) was used to determine pERK1/2 and total ERK1/2 expression compared to HSP70 loading controls, *n* = 3. Error bars = SEM, One-Way ANOVA with Bonferroni corrections, *p < 0.05, **p < 0.01, ***p < 0.001, ****p < 0.0001. Blots have been cropped for clarity and conciseness. Full-length blots are presented in Supplementary Figure S1b

Our findings have shown subtype-specific biological effects of EVs derived from different clonal sources of MSCs, which may be linked to the intra- and extra-vesicular protein composition and uptake mechanisms by target cells. Considering our evidence for an enriched ECM-based corona in Y201 EVs, particularly the abundance of RGD-containing proteins (for example, FN1 and MFG-E8), we hypothesised that these EV subtypes were preferentially taken up by integrin-mediated endocytosis. Y202 cells were used as model target cells and treated with GRGDSP integrin blocking peptides or GRADSP peptide controls for 6 h followed by exposure to CFSE-stained Y201 EVs. Using flow cytometry, we demonstrated that Y201 EV uptake was significantly inhibited by RGD blockade compared to controls (Fig. 10A, B). Focal adhesion kinase (FAK) acts downstream of RGD integrins to mediate outside-in signalling through phosphorylation of ERK1/2 [48]. We demonstrated by western blot analysis that Y201 EVs stimulated phosphorylation of ERK1/2 after 30 min of exposure in Y202 cells, and that this effect was mediated by FAK (Fig. 10C–F).

**Fig. 11:**
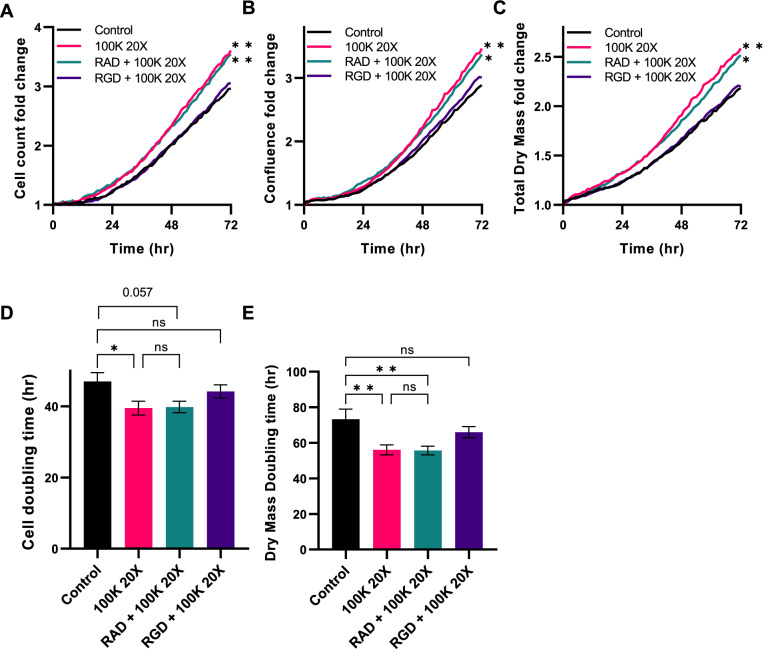
Effect of Y201 EVs on proliferation of primary articular chondrocytes, with and without RGD blocking peptides (GRGDSP) or RAD control peptides (GRADSP). **A** Cell counts, **B** Confluence, **C** Total dry mass, **D** cell doubling time and **E** dry mass doubling time of chondrocytes. *n* = 3, Error bars = SEM, Two-Way or One-Way ANOVA, ,*p < 0.05, **p < 0.01, ns = not significant

To confirm the role of RGD integrins in Y201 EV bioactivity, primary ACs were treated with GRGDSP (and the GRADSP control peptide), and the effect of Y201 EVs on their proliferation rate was monitored for 72 h. We confirmed that Y201 EVs increased AC proliferation compared to untreated controls and that this effect was blocked by exposure to GRGDSP but not GRADSP peptides (Fig. 11).

### Effect of Y201 EVs and Y202 EVs on T cell function

**Fig. 12:**
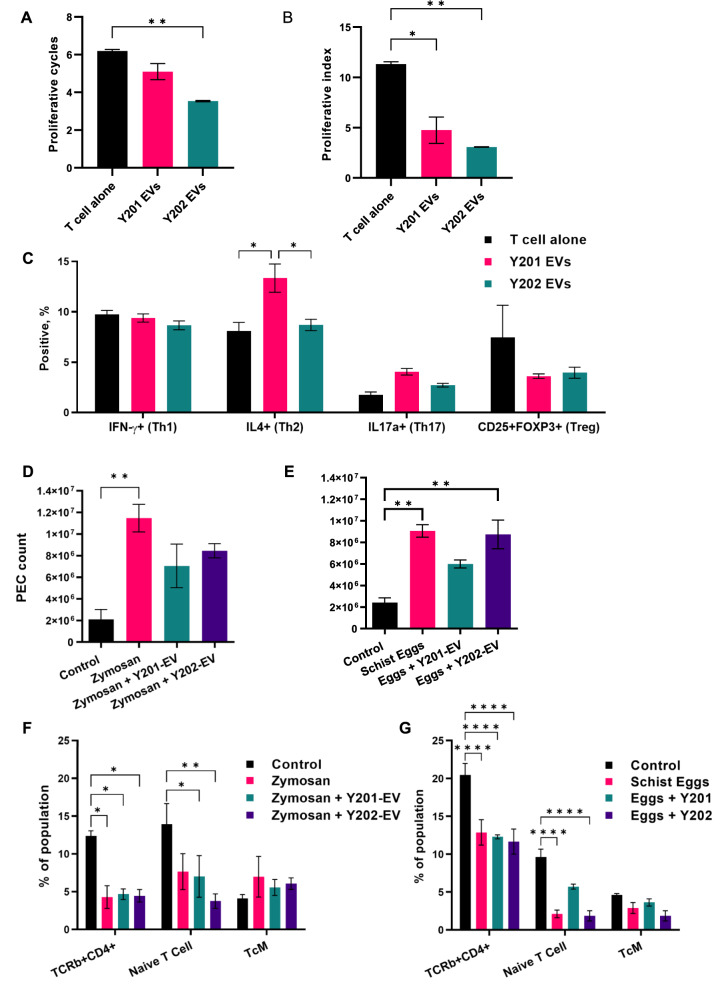
Immunomodulatory capacity of EVs isolated from Y201 and Y202 MSC subtypes. In vitro treatment of activated CD4+ T cells with Y201 and Y202 EVs (*n* = 2, mean events > 18,000 counted) **A** Proliferative cycles. **B** Proliferative index **C** Polarisation of activated T cells in the absence and presence of Y201 EVs and Y202 EVs (*n* = 2, mean events > 32,000 counted). D/E) Total peritoneal exudate cell (PEC) counts following zymosan (**D**) or schistosome egg (**E**) induced inflammation in the absence and presence of Y201 EVs and Y202 EVs. **F**, **G** Examination of TCR+ CD4+, naïve and central memory T cells in zymosan or schistosome egg induced inflammation in the absence and presence of Y201 EVs and Y202 EVs (*n* = 3, 12 per experiment, total 24). One-Way ANOVA with Bonferroni post hoc testing, *p < 0.05, **p < 0.01, ***p < 0.001

A frequently reported immune function of MSCs is to suppress activated T cell proliferation. Here, T cell proliferation was assessed by determining gradual division (proliferative index) and population doublings (proliferative cycles) over 5 days of co-culture with or without Y201 EVs and Y202 EVs. Compared to T cells alone, both EV subsets significantly reduced proliferative index scores and Y202 EVs reduced proliferative cycles (Fig. 12A, B). Next, we determined the influence of Y201 EVs and Y202 EVs on the polarisation of naïve T cells into effector lineages with immunosuppressive/anti-inflammatory function. Y201 EVs, but not Y202 EVs, caused a significant increase in the development of anti-inflammatory Th2 cells, as indicated by intracellular IL4 staining (Fig. 12C). There were no significant effects on Th1 Th17 and Treg cell numbers, as indicated by IFN-γ, IL17a and CD25/FOXP3 expression respectively (Fig. 12C).

### In vivo assessment of immunomodulatory capacity of Y201 and Y202 EVs in a murine peritonitis model

Immune regulation was evaluated by Y201 and Y202 MSCs in zymosan- and schistosome egg-induced peritonitis model of acute inflammation that promotes the recruitment of monocytes and neutrophils to the peritoneal cavity. Following treatment with irritant, peritoneal exudate cells (PEC) were collected by lavage and analysis performed on the cell content. A gating strategy was devised for flow cytometric analysis of multiple PEC cell types focusing on haematopoietic, myeloid and lymphoid cells including monocytes, macrophages and T cells. In contrast to the zymosan only treatment group (11.5 ± 1.3 × 10^6^ PECs), treatment with EVs from either Y201 (7.1 ± 2.0 × 10^6^ PECs) or Y202 (8.5 ± 0.7 × 10^6^ PECs) MSC lines did not significantly increase the number of PEC cells in comparison to PBS controls (2.1 ± 1.6 × 10^6^ cells) (Fig. 12D). When schistosome egg irritant was applied, both untreated irritant (9.1 ± 0.6 × 10^6^ cells) and Y202 EVs treatment (8.7 ± 1.3 × 10^6^ cells) showed significant recruitment of immune cells to the peritoneal cavity over PBS controls (2.4 ± 0.4 × 10^6^ PECs) whilst Y201 EVs (6.0 ± 0.4 × 10^6^ PECs) suppressed increases observed in PEC counts (Fig. 12E). It was notable that using the schistosome egg-induced peritonitis model, but not zymosan, Y201 EV were able to maintain naive T cell numbers at levels significantly higher than egg-treatment only or Y202 EVs (Fig. 12F, G). Similarly, Y201 EV treatment dampened the effects of the irritant on myeloid cell recruitment to the region of inflammation, typically seen in acute *Schistosoma mansoni* infection. In particular, data indicated suppression of macrophage and neutrophil recruitment to the peritoneal cavity in response to schistosome egg-induced peritonitis (Supplementary material 2: Supplementary Fig. S9).

### In vivo assessment of potential therapeutic efficacy of Y201 and Y202 EVs in a murine arthritis model

**Fig. 13:**
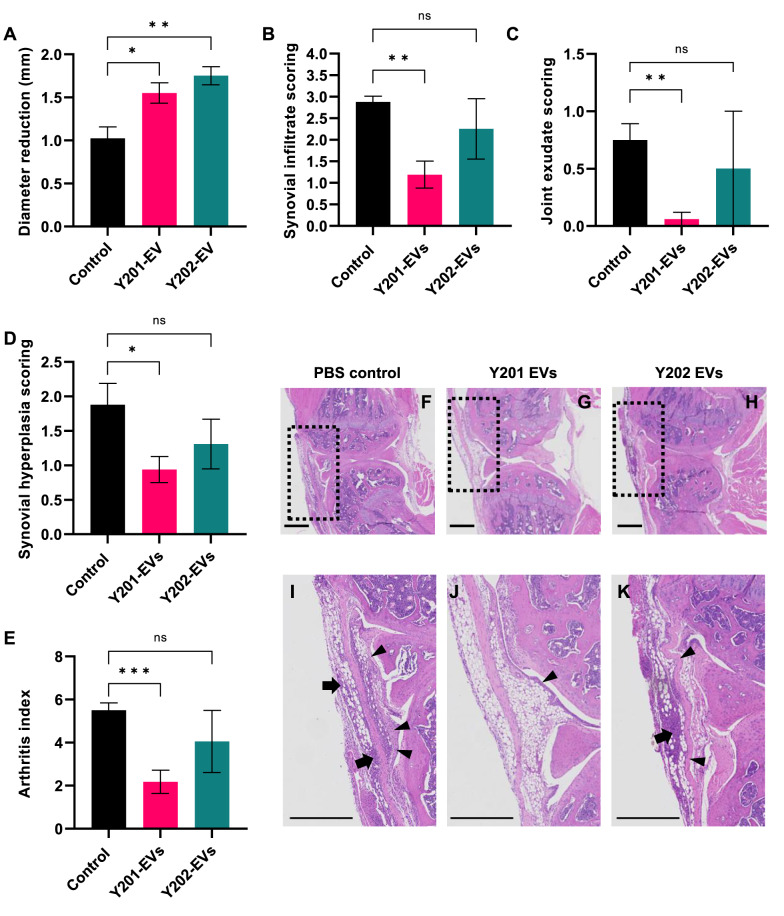
Effect of Y201 and Y202 EVs on disease activity in an in vivo antigen-induced arthritis model. **A** Joint size reduction from peak swelling (mm), **B** synovial infiltrate score, **C** joint exudate score, **D** synovial hyperplasia score, **E** arthritis index. Haematoxylin and eosin-stained sections of **F** and **I** PBS control, **G** and **J** Y201 EV-treated mouse knee joints, and **H** and **K** Y202 EV-treated mouse knee joints. Boxed areas in **F**, **G** and **H** are enlarged in **I**, **J** and **K** respectively showing examples of synovial infiltrate (cellular infiltration into synovium; arrows) and synovial hyperplasia (thickened synovial lining; arrowheads). Scale bars = 500 μm. Mean values shown ± SD (*n* = 4) Brown-Forsythe ANOVA test, *p < 0.05, **p < 0.01, ***p < 0.001, ns = not significant. The total number of animals used = 12

Finally, we tested the bioactivities of Y201 and Y202 EVs in a disease-relevant in vivo model of antigen-induced arthritis (AIA) model following intra-articular EV injection. Following histological examination and blind scoring, we demonstrated that EVs derived from Y201 MSCs induced a significant decrease in all measures of disease activity compared to vehicle controls, including joint swelling, synovial infiltrate, joint exudate, synovial hyperplasia and overall arthritis index (Fig. 13A–E). Y202 EVs significantly reduced joint swelling compared to controls but otherwise did not affect any other disease score measures. Representative haematoxylin and eosin-stained sections provide evidence of changes in synovial infiltrate and hyperplasia in control, Y201 EV and Y202 EV-treated samples (Fig. 13F–K).

## Discussion

There is a growing interest in the therapeutic application of EVs derived from different cell sources and MSCs in particular. It is clear that EV composition and bioactivity is determined to a large extent by the biology of the parent cell [49–52] and MSC tissue source [53, 54]. Here we have demonstrated that stromal cell lines, clonally isolated from the same mixed donor population of MSCs, have distinct features and functionalities. These findings are important as almost all basic research, preclinical and clinical studies employ EVs from heterogeneous MSC pools, which we can now confirm will be prone to significant within-population variability. Moreover, the analysis of EVs derived from two related MSC subtypes with different bioactivities also assisted the identification of key functional EV features, which has informed MSC EV mechanism of action.

It was notable that the repertoire of lipids, miRNAs and proteins presented by Y201- and Y202-derived EVs was remarkably similar and may contribute to defining a common stromal EV biomarker signature. More in-depth analyses demonstrated differences in the relative abundance of these EV components, particularly within the EV proteome, where Y201 EVs were found to be significantly enriched compared to Y202 EVs. Numerous studies have analysed the MSC-EV proteome with a diverse array of outcomes, comprehensively reviewed by Qiu and colleagues [55]. A detailed interrogation of published reports has described an MSC-EV-specific signature, by comparing 10 MSC-EV datasets with 12 non-MSC-EV datasets [56]. The signature includes a number of proteins we identified, including collagen I and VI, filamins, actin and vimentin, but noticeably absent were FN1 and MFG-E8, which are the two most abundant Y201-EV proteins. Although the Y201-EVome is varied and complex, the enrichment of RGD-containing FN1 and MFG-E8 may be a defining feature that directly contributes to their bioactivity. Bioinformatic interrogation of our own datasets demonstrated that Y201 EV proteins predominantly contribute to matrix organisation, cell-cell and cell-matrix interactions as well as immune regulation. We hypothesise that a significant proportion of the identified proteins contribute to an EV corona, presented on the exofacial surface by integrins and other adhesion molecules, though further experimental validation is required. There is a growing understanding of the influential contribution the corona makes to EV function [57–61]. Recent work has shown that EV-delivered cargoes were only detectable in recipient cells at high EV doses and that the overriding biological effects were mediated by EV surface proteins at lower EV doses that more closely represented physiological conditions [51].

When comparing Y201 EVs with Y202 EVs, we attempted to consider physiologically-relevant effects by adopting a dosing strategy that accounted for EV yield per cell (Y201 MSCs produce significantly more EVs per cell than Y202 MSCs), though we recognise that improved attempts to define EV dose in this study and others are required. We were able to demonstrate that at least some of the effects of Y201 EVs were mediated via an RGD (integrin)-FAK-ERK1/2 axis, with increases in pERK1/2 detected within 30 min of exposure. Similar observations have been reported in other cell types [62–64] though the vast majority of studies focus on EV cargo delivery as a means of mediating biological effect. Here, we showed that Y201 EV internalisation, and potentially therefore cargo delivery, took 2–4 h. These findings suggest that target cell responses will be determined not only by biological make-up of EVs presented to the cell surface, but also the cell’s capacity for EV uptake.

The precise mechanisms by which Y201 EVs exert anti-inflammatory effects in vitro and in vivo are less clear, though MFG-E8 is an interesting candidate. MFG-E8 (also known as lactadherin) is an anti-inflammatory glycoprotein, enriched in the milk fat globule membrane (MFGM) where it contributes to its immune-boosting and antipathogenic activity to protect newborns [65]. Interestingly, lactosylceramide, which was significantly enriched in Y201 EV lipids, is also abundant in the MFGM [66]. MFG-E8 has protective effects in rheumatoid arthritis and other inflammatory conditions [67–70]. MFG-E8 can also restore chondrocyte function, enhance osteogenesis and reduce inflammatory bone loss in osteoarthritis [71–73]. It is tempting to speculate that Y201 EVs mimic an apoptosis-induced anti-inflammatory response whereby Y201 EVs, like apoptotic cells, present MFG-E8 and other regulatory molecules to immune cells to promote cell clearance and suppress inflammation [67]. Intriguingly, our findings may explain why the immunosuppressive effects of infused MSCs appear to be mediated by MSC apoptosis [11]. However, further work that embraces the biological complexity of EVs is clearly needed; given the plethora of other proteins we identified, including for example HSPG2, THBS1, BGN, NID1 and VTN, the mechanism of action of Y201 EVs is likely to be multi-factorial.

## Conclusions

EVs released by different MSC subtypes differ significantly in terms of cargo, biomarker presentation, bioactivity and pre-clinical in vivo efficacy. Consequently, heterogeneity within a non-clonal MSC population will affect the therapeutic value of the collective EV pool they produce. Detailed analysis of defined MSC and EV subsets will advance our understanding of their mechanisms of action and enable real progress in clinical development for targeted indications. Current evidence suggests that EVs derived from a population of Y201-type MSCs would have high therapeutic value in the treatment of inflammatory disorders associated with tissue loss.

## Supplementary Information

Below is the link to the electronic supplementary material.

Supplementary Material 1.

Supplementary Material 2.

Supplementary Material 3.

## Data Availability

Raw data files and data tables in .csv format including descriptions of samples and their associated files, and lipid annotations for all detected lipids and associated feature areas across files, have been uploaded to MassIVE, dataset MSV000097745. All other data generated or analysed during this study are included in this published article and its supplementary information files.

## Abbreviations

AC: Articular chondrocyte
AIA: Antigen-induced arthritis
BiP: Binding immunoglobulin protein
BSA: Bovine serum albumin
CFSE: Carboxyfluorescein diacetate succinimidyl-ester
DMEM: Dulbecco’s Modified Eagle’s Medium
ECM: Extracellular matrix
EV: Extracellular vesicle
FAK: Focal adhesion kinase
FAKi: Focal adhesion kinase inhibitor
FBS: Foetal Bovine Serum
FDR: False discovery rate
FN1: Fibronectin
GO: Gene ontology
KEGG: Kyoto Encyclopedia of Genes and Genomes
LC-MS/MS: Liquid chromatography with tandem mass spectrometry
MFG-E8: Milk fat globule epidermal growth factor 8
MFGM: Milk fat globule membrane
miRNAs: MicroRNAs
MSC: Mesenchymal stromal cell
MV: Microvesicle
MVB: Multivesicular body
NTA: Nanoparticle tracking analysis
P/S: Penicillin/streptomycin
PBS: Phosphate buffered saline
PEC: Peritoneal exudate cells
TEM: Transmission electron microscopy
