# Supplementary material for: Extracellular vesicle bioactivity and potential for clinical development are determined by mesenchymal stromal cell clonal subtype
Source: Stem Cell Res Ther. 2025 Oct 16;16:571. doi: 10.1186/s13287-025-04665-z (PMC12532472; doi:10.1186/s13287-025-04665-z)
Supplement: Supplementary file 1 — Supplementary Material 1. [file 13287_2025_4665_MOESM1_ESM.pdf]

NOTE: Please save this file locally before filling in the table, DO NOT work on the file within your internet browser as changes will not be saved. Adobe Acrobat Reader (available free here) is recommended for completion.

The ARRIVE guidelines 2.0: author checklist

The ARRIVE Essential 10

These items are the basic minimum to include in a manuscript. Without this information, readers and reviewers cannot assess the reliability of the findings.

Item		Recommendation	Section/line number, or reason for not reporting
**Study design**	1	For each experiment, provide brief details of study design including:	Peritonitis:
		a. The groups being compared, including control groups. If no control group has been used, the rationale should be stated.	a) 403-407
		b. The experimental unit (e.g. a single animal, litter, or cage of animals).	b) 396
**Sample size**	2	a. Specify the exact number of experimental units allocated to each group, and the total number in each experiment. Also indicate the total number of animals used.	405-407, 1184
		b. Explain how the sample size was decided. Provide details of any *a priori* sample size calculation, if done.	Sample size based on our prior study
**Inclusion and exclusion criteria**	3	a. Describe any criteria used for including and excluding animals (or experimental units) during the experiment, and data points during the analysis. Specify if these criteria were established *a priori*. If no criteria were set, state this explicitly.	Animals were checked a priori for good health.
		b. For each experimental group, report any animals, experimental units or data points not included in the analysis and explain why. If there were no exclusions, state so.	No exclusions were made.
		c. For each analysis, report the exact value of *n* in each experimental group.	405-407, 1184
**Randomisation**	4	a. State whether randomisation was used to allocate experimental units to control and treatment groups. If done, provide the method used to generate the randomisation sequence.	400
		b. Describe the strategy used to minimise potential confounders such as the order of treatments and measurements, or animal/cage location. If confounders were not controlled, state this explicitly.	401
**Blinding**	5	Describe who was aware of the group allocation at the different stages of the experiment (during the allocation, the conduct of the experiment, the outcome assessment, and the data analysis).	The experimenters were blinded while conducting the experiment and data analysis
**Outcome measures**	6	a. Clearly define all outcome measures assessed (e.g. cell death, molecular markers, or behavioural changes).	412-417, 676-679
		b. For hypothesis-testing studies, specify the primary outcome measure, i.e. the outcome measure that was used to determine the sample size.	N/A
**Statistical methods**	7	a. Provide details of the statistical methods used for each analysis, including software used.	443-448, 1184-1185
		b. Describe any methods used to assess whether the data met the assumptions of the statistical approach, and what was done if the assumptions were not met.	443-448
**Experimental animals**	8	a. Provide species-appropriate details of the animals used, including species, strain and substrain, sex, age or developmental stage, and, if relevant, weight.	396-397
		b. Provide further relevant information on the provenance of animals, health/immune status, genetic modification status, genotype, and any previous procedures.	396-397
**Experimental procedures**	9	For each experimental group, including controls, describe the procedures in enough detail to allow others to replicate them, including:	400-417
		a. What was done, how it was done and what was used.	401-407
		b. When and how often.	400-417, 804-806
		c. Where (including detail of any acclimatisation periods).	
		d. Why (provide rationale for procedures).	673-675
**Results**	10	For each experiment conducted, including independent replications, report:	
		a. Summary/descriptive statistics for each experimental group, with a measure of variability where applicable (e.g. mean and SD, or median and range).	673-690, Figure 12
		b. If applicable, the effect size with a confidence interval.	673-690, Figure 12

NOTE: Please save this file locally before filling in the table, DO NOT work on the file within your internet browser as changes will not be saved. Adobe Acrobat Reader (available free here) is recommended for completion.

The ARRIVE guidelines 2.0: author checklist

The ARRIVE Essential 10

These items are the basic minimum to include in a manuscript. Without this information, readers and reviewers cannot assess the reliability of the findings.

Item		Recommendation	Section/line number, or reason for not reporting
**Study design**	1	For each experiment, provide brief details of study design including:	**AIA study:**
		a. The groups being compared, including control groups. If no control group has been used, the rationale should be stated.	Line 422-424
		b. The experimental unit (e.g. a single animal, litter, or cage of animals).	Line 420
**Sample size**	2	a. Specify the exact number of experimental units allocated to each group, and the total number in each experiment. Also indicate the total number of animals used.	Line 424<br>Line 1195-1196
		b. Explain how the sample size was decided. Provide details of any *a priori* sample size calculation, if done.	Sample size was decided based on our previous experince with this model (AIA)
**Inclusion and exclusion criteria**	3	a. Describe any criteria used for including and excluding animals (or experimental units) during the experiment, and data points during the analysis. Specify if these criteria were established *a priori*. If no criteria were set, state this explicitly.	No animals or data points were excluded during the experiment or analysis;
		b. For each experimental group, report any animals, experimental units or data points not included in the analysis and explain why. If there were no exclusions, state so.	No animals have been ruled out;
		c. For each analysis, report the exact value of *n* in each experimental group.	Line 424 & 1195
**Randomisation**	4	a. State whether randomisation was used to allocate experimental units to control and treatment groups. If done, provide the method used to generate the randomisation sequence.	Line 432
		b. Describe the strategy used to minimise potential confounders such as the order of treatments and measurements, or animal/cage location. If confounders were not controlled, state this explicitly.	Confounders were not controlled
**Blinding**	5	Describe who was aware of the group allocation at the different stages of the experiment (during the allocation, the conduct of the experiment, the outcome assessment, and the data analysis).	The experimenters were blinded while conducting the experiment and assessing the outcome
**Outcome measures**	6	a. Clearly define all outcome measures assessed (e.g. cell death, molecular markers, or behavioural changes).	Line 430-433<br>Line 437-440
		b. For hypothesis-testing studies, specify the primary outcome measure, i.e. the outcome measure that was used to determine the sample size.	N/A
**Statistical methods**	7	a. Provide details of the statistical methods used for each analysis, including software used.	Line 475-479; line 1195
		b. Describe any methods used to assess whether the data met the assumptions of the statistical approach, and what was done if the assumptions were not met.	Line 475-479
**Experimental animals**	8	a. Provide species-appropriate details of the animals used, including species, strain and substrain, sex, age or developmental stage, and, if relevant, weight.	Line 420-421
		b. Provide further relevant information on the provenance of animals, health/immune status, genetic modification status, genotype, and any previous procedures.	Line 420-421
**Experimental procedures**	9	For each experimental group, including controls, describe the procedures in enough detail to allow others to replicate them, including:	
		a. What was done, how it was done and what was used.	Line 420-430
		b. When and how often.	Line 422-431
		c. Where (including detail of any acclimatisation periods).	Line 806-809
		d. Why (provide rationale for procedures).	Line 48-50
**Results**	10	For each experiment conducted, including independent replications, report:	Line 475-479<br>Line 1195-1196
		a. Summary/descriptive statistics for each experimental group, with a measure of variability where applicable (e.g. mean and SD, or median and range).	Figure 13
		b. If applicable, the effect size with a confidence interval.	Figure 13 (A-E)
